# OsCAF2 contains two CRM domains and is necessary for chloroplast development in rice

**DOI:** 10.1186/s12870-020-02593-z

**Published:** 2020-08-18

**Authors:** Lan Shen, Qiang Zhang, Zhongwei Wang, Hongling Wen, Guanglian Hu, Deyong Ren, Jiang Hu, Li Zhu, Zhenyu Gao, Guangheng Zhang, Longbiao Guo, Dali Zeng, Qian Qian

**Affiliations:** 1 State Key Laboratory of Rice Biology / China National Rice Research Institute, Chinese Academy of Agricultural Sciences, Hangzhou, 310006 China; 2 Biotechnology Research Center, Chongqing Academy of Agricultural Sciences, Chongqing, 401329 China

**Keywords:** *OsCAF2*, CRM domain, Intron splicing, Chloroplast development, Rice

## Abstract

**Background:**

Chloroplasts play an important role in plant growth and development. The chloroplast genome contains approximately twenty group II introns that are spliced due to proteins encoded by nuclear genes. CAF2 is one of these splicing factors that has been shown to splice group IIB introns in maize and *Arabidopsis thaliana.* However, the research of the *OsCAF2* gene in rice is very little, and the effects of *OsCAF2* genes on chloroplasts development are not well characterized.

**Results:**

In this study, *oscaf2* mutants were obtained by editing the *OsCAF2* gene in the *Nipponbare* variety of rice. Phenotypic analysis showed that mutations to *OsCAF2* led to albino leaves at the seeding stage that eventually caused plant death, and *oscaf2* mutant plants had fewer chloroplasts and damaged chloroplast structure. We speculated that *OsCAF2* might participate in the splicing of group IIA and IIB introns, which differs from its orthologs in *A. thaliana* and maize. Through yeast two-hybrid experiments, we found that the C-terminal region of OsCAF2 interacted with OsCRS2 and formed an OsCAF2-OsCRS2 complex. In addition, the N-terminal region of OsCAF2 interacted with itself to form homodimers.

**Conclusion:**

Taken together, this study improved our understanding of the OsCAF2 protein, and revealed additional information about the molecular mechanism of OsCAF2 in regulating of chloroplast development in rice.

## Background

Chloroplasts are necessary organelles for plant growth and development, as they biosynthesize carbohydrates through the fixation of CO_2_ [1, 2]. Chloroplasts are derived from proplastids and are semiautonomous organelles [3]. The chloroplast genome is small and encodes approximately 100 proteins responsible largely for photosynthesis, carbon metabolism, and fatty acid synthesis [4]. Many studies have indicated that plastid-encoded polymerases (PEPs) and nucleus-encoded polymerases (NEPs) play key roles in regulating chloroplast development. These studies suggest that expressions of chloroplast genes require proteins encoded by the nuclear genome [5, 6]. These proteins are involved in the transcription and post-transcription modification of chloroplast related genes, such as chloroplast DNA replication, RNA transcription, RNA editing, RNA stability, and RNA splicing [6].

The process of RNA splicing occurs by removing introns from the precursor messenger mRNA (pre-mRNA) to join adjacent exons [7]. In plants, the introns of chloroplast genes are divided into two groups, known as group I and group II introns, based on their conserved primary and secondary structures, as well as the splicing mechanism of RNA splicing. Group II introns are further divided into two subgroups, subgroup IIA and subgroup IIB [7, 8]. Previous work has shown that group I and group II introns of chloroplast genes have lost the ability for autocatalytic splicing, and the introns of these genes lose elements that were necessary for RNA-based catalytic centers [8, 9]. Chloroplast RNA splicing requires the recruitment of proteins that are encoded by nuclear genes. These proteins act as splicing factors that are essential for chloroplast RNA splicing reactions [7, 10]. In *Arabidopsis thaliana*, chloroplast genome contains 20 group II introns and 1 group I intron. In maize and rice, the chloroplast genome consists of 17 group II introns and one group I intron [11]. It has been reported that many nuclear genes encode proteins that act as splicing factors that are involved in the regulation of chloroplast RNA splicing. For example, pentatricopeptide repeat (PPR) proteins and chloroplast RNA splicing and ribosome maturation (CRM) domain proteins are involved in the regulation of splicing of chloroplast gene introns [7].

Previous studies also showed that many PPR proteins participate in the splicing of group II introns in various plant species [12, 13]. For example, in maize, ZmPPR4 and ZmPPR5 promote intron splicing of *rps12* and *trnG* in the chloroplast [14, 15]. In *A. thaliana*, AtOTP70 and AtOTP51 are required for intron splicing of *rpoC1* and *ycf3–2* [16, 17]. In rice, WSL4 and PGL12 participate in intron splicing of *rpl2* and *ndhA*, respectively [18, 19]. Inactivation of the PPR proteins involved in chloroplast intron splicing leads to abnormal chloroplast development, with leaves showing chlorosis or albino seedling phenotypes [20, 21].

With the exception of PPR proteins, some CRM domain-containing proteins act as splicing factors that regulate the splicing of chloroplast introns. It has been reported that the CRM domain-containing proteins influence chloroplast development in *A. thaliana,* maize, and rice [7]. The first CRM gene, *CRS1*, was identified and cloned in maize. ZmCRS1 contains three CRM domains, which have high affinity and specificity in vitro for the *atpF* intron, and they participate in intron splicing of *atpF* [9, 22]. ZmCAF1 and ZmCAF2 contain two CRM domains, and each of them can interact with ZmCRS2 and form ZmCAF1-ZmCRS2 and ZmCAF2-ZmCRS2 complexes [23]. The ZmCAF1-ZmCRS2 complex is required for the splicing of subgroup IIB introns, including *rpl16*, *rps16*, *trnG*, *petD*, and *ycf3* [24]. In *A. thaliana,* the AtCAF1-AtCRS2 complex also promoted the splicing of the *rpoC1* and *ClpP* introns, which were absent in the maize chloroplast genome [25]. Furthermore, earlier studies showed that the C-terminal region of ZmCAF2 can interact with ZmCRS2 to form the ZmCAF2-ZmCRS2 complex, which was required for splicing of five subgroup IIB introns from the genes *ndhA*, *ndhB*, *rps12*, *petB*, and *ycf3* [23, 24]. The CFM2 has four CRM domains. In maize, ZmCFM2 was required to splice the *trnL*, *ndhA*, and *ycf3* introns [26]. In *A. thaliana*, AtCFM2 can splice the *trnL*, *ndhA*, and *ycf3* introns, and it influences splicing of the *clpP* intron that was absent from the maize chloroplast genome [26]. Unlike other proteins contained in the CRM domains, CFM3 contains three CRM domains and was located in both chloroplasts and mitochondria. In *A. thaliana*, AtCFM3a was necessary for splicing of the *ndhB* intron [26]. In rice, fourteen proteins containing one or more CRM domains have been identified [7]. The *oscfm3* mutant resulted in an albino phenotype in seedlings caused by abnormal chloroplast development. OsCFM3 promotes splicing of chloroplast genes *ndhB*, *petD*, and *rps16* [26]. Previous research showed that *AL2* encodes OsCRS1, which is homologous to AtCRS1 and ZmCRS1, and contains three CRM domains. However, OsCRS1 promotes splicing of group I (*trnL*) and group II introns (*atpF*, *rpl2*, *ndhA*, *ndhB*, *petD*, and *ycf3*) that differ from the homologous proteins AtCRS1 and ZmCRS1 [11]. It has been reported that OsCAF1 contains two CRM domains that could interact with OsCRS2 via its C-terminal region and forms the OsCAF1-OsCRS2 complex in rice. The OsCAF1 localizes to the chloroplast and affects splicing of subgroup IIA and subgroup IIB introns [27]. The function of other proteins containing the CRM domain has not been studied extensively in rice.

In this study, we generated an *oscaf2* mutant by using the CRISPR/Cas9 (Clustered Regularly Interspaced Short Palindromic Repeats) gene-editing system and studied the role of OsCAF2 in rice chloroplast development. Our results showed that mutants of *OsCAF2* lead to abnormal chloroplast development and albino phenotypes in seedlings. Analysis of intron splicing of chloroplast genes showed that OsCAF2 promoted the splicing of chloroplast subgroup IIA and subgroup IIB introns, which were necessary for chloroplast development. Similar to the ZmCAF2-ZmCRS2 complex, the C-terminal region of OsCAF2 interacted with OsCRS2 and formed the OsCAF2-OsCRS2 complex. Interestingly, OsCAF2 also could interact with itself through its N-terminal region to form dimers that regulate chloroplast development in rice. These results demonstrate the molecular mechanism of OsCAF2 in rice chloroplast development.

## Results

### Disruption of OsCAF2 function caused an albino phenotype

**Fig. 1:**
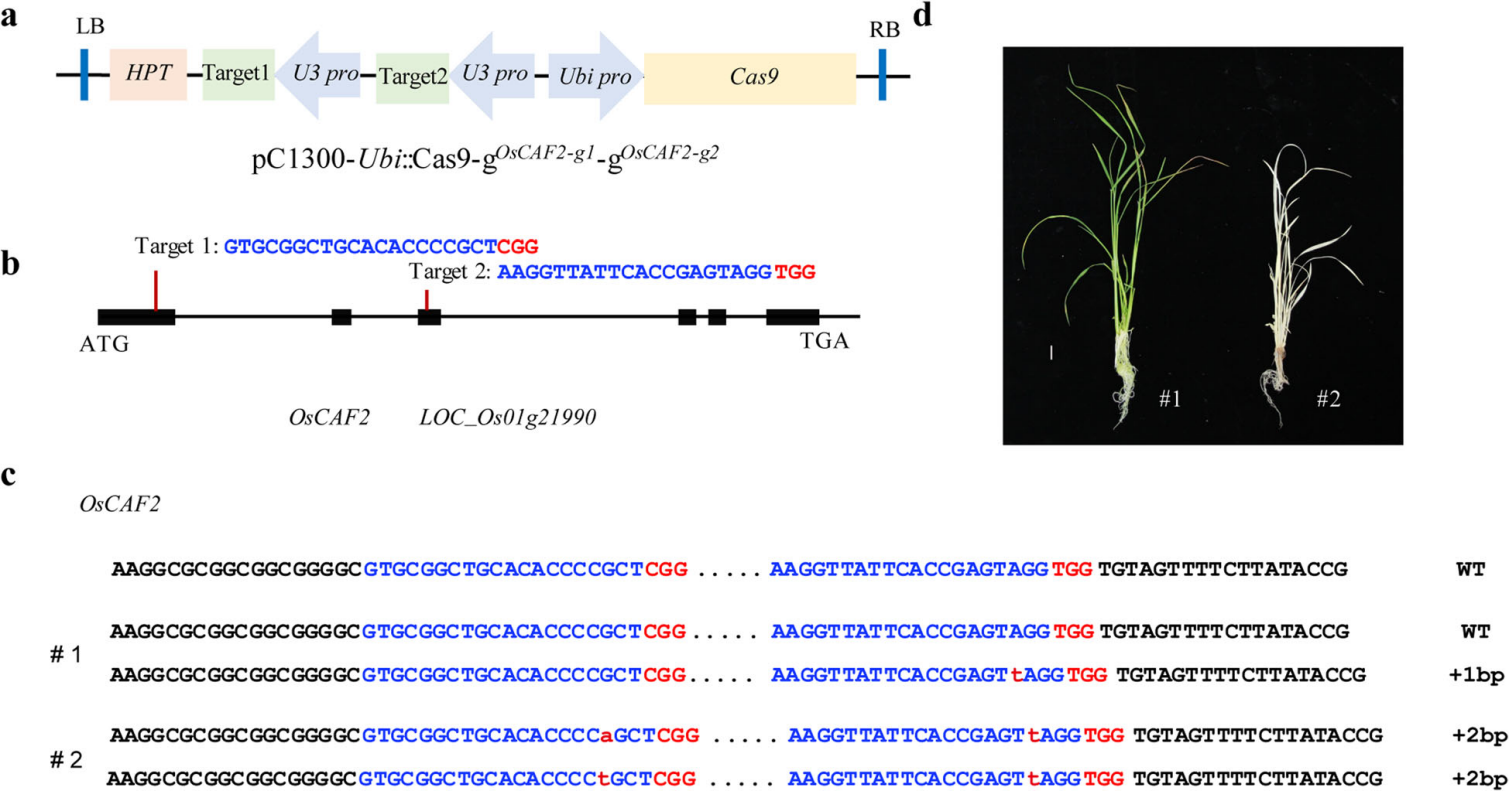
Disruption of OsCAF2 results in an albino phenotype. **a** Schematic diagram of CRISPR/Cas9 system for editing OsCAF2. **b** Diagram of the targeted site in OsCAF2. **c** Mutation types of transformed plants in the T_0_ generation. The protospacer adjacent motif sequence is shown in red. The targeted sequences are shown in blue. **d** The phenotype of the two positive plants at the seedling stage. Scale bar = 1 cm

**Fig. 2:**
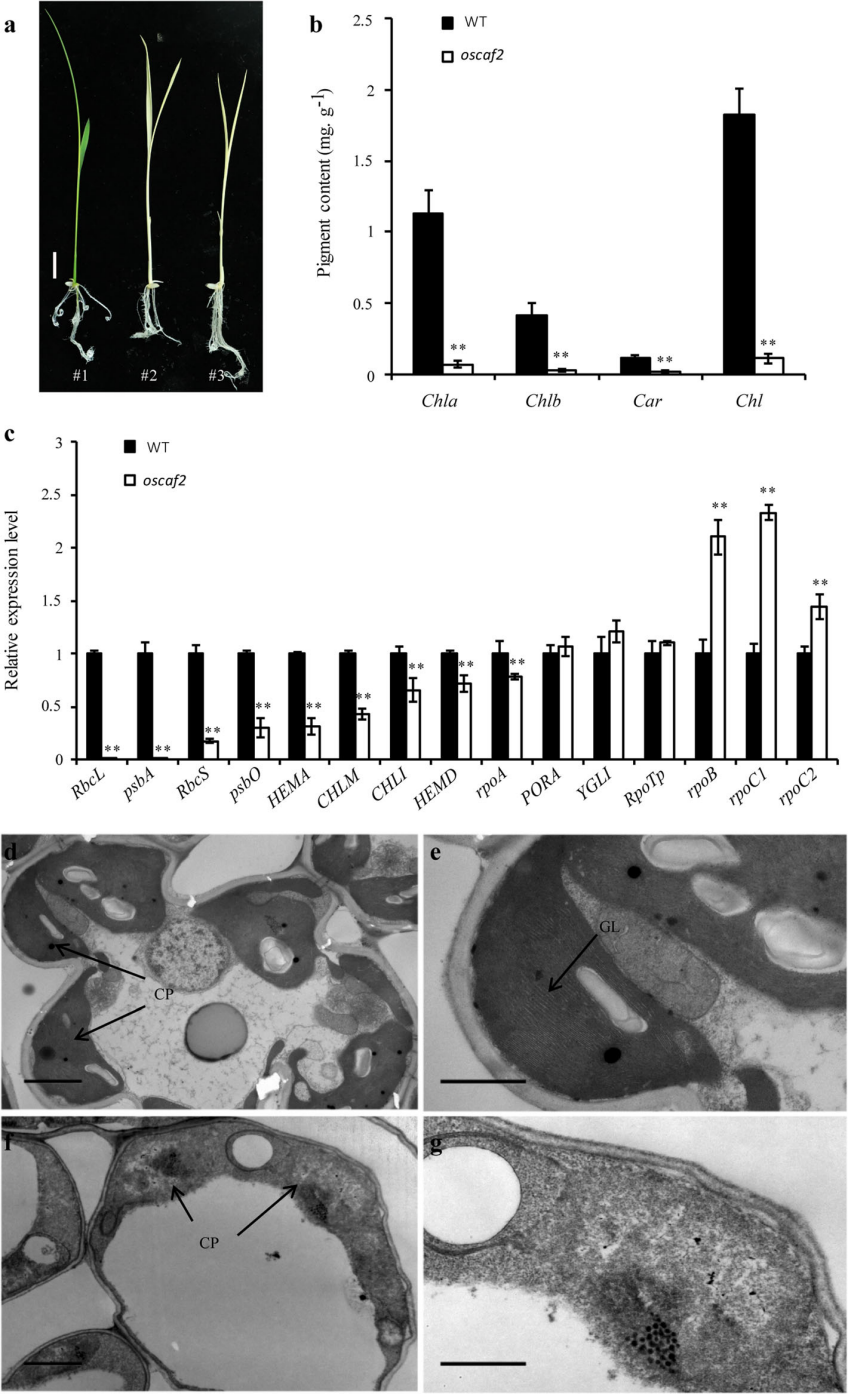
Chloroplast development was impaired in the *oscaf2* mutant. **a** Phenotype presentation of WT (#1) and *oscaf2* mutant (#2 and #3). Scale bar = 1 cm. **b** Chlorophyll contents of WT and *oscaf2* at the two leaf stage. **c** Expression analysis of chloroplast synthesis and photosynthesis-related genes in WT and the *oscaf2* mutant. The values represent the mean of three independent experiments. Error bars indicate SE, ** indicate *P* < 0.01, respectively. **d-g** The chloroplast ultrastructure of WT and *oscaf2* mutants from leaves. WT **(d, e)** and *oscaf2* mutants **(f, g)**. CP, chloroplast, GL, grana lamella. Scale bars of **d** and **f**, 2 μm, Scale bars of **e** and **g**, 1 μm

According to previous studies, the gene homologous to *ZmCAF2* and *AtCAF2* from maize and *A. thaliana* was identified from the rice genome and named *OsCAF2* (*LOC_Os01g21990*) [7]. The *OsCAF2* contains six exons, with a total coding region of 1824 bp, which encodes 608 amino acids. The OsCAF2 contains two CRM domains, but their functions are unknown. To study the function of OsCAF2, we constructed the *OsCAF2* gene editing vector including two target sgRNAs in exon 1 (upstream of the two CRM domains) and exon 3 (the first CRM domain) respectively, and transformed it into *Nipponbare* via the *Agrobacterium* method (Fig. 1a-b). In the T_0_ generation, we obtained 15 plants. Through PCR amplification and sequencing of *OsCAF2*, we screened one homozygous mutants and one heterozygote plant, named *oscaf2* and *oscaf2/OsCAF2,* respectively (Fig. 1c). The mutation types of one or two base insertions lead to the premature termination of OsCAF2 translation. Phenotypic observations suggested that the *oscaf2* mutants showed albino seedling phenotypes and died at the three leaf stage, but the heterozygote *oscaf2/OsCAF2* showed green leaves and normal growth (Fig. 1d). To investigate the function of OsCAF2, we obtained *oscaf2* mutants in the T_1_ generation of *oscaf2/OsCAF2* heterozygote plant (Fig. 2a). The *oscaf2* mutants from *oscaf2/OsCAF2* heterozygote plant also appeared as albino and then died at the three leaf stage (Fig. 2a). Compared to wild type (WT), the Chl a, Chl b, car and Chl content in *oscaf2* mutant leaves were significantly decreased, and was practically devoid of car and Chl b (Fig. 2b). These results suggest that *OsCAF2* was necessary for chloroplast development, and that mutation of *OsCAF2* resulted in an albino phenotype in seedlings.

### Expression analysis of chloroplast development and photosynthesis-related genes in *oscaf2* mutants

To further analysis the effects of impaired chloroplast development in the *oscaf2* mutant, we measured the expression levels of chloroplast development and photosynthesis-related genes. PEPs and NEPs are regulated transcriptionally by chloroplast-encoded genes responsible for chloroplast development. Expression levels of the NEP component gene *OsRpoTp* in the *oscaf2* mutant was normal compared to the WT (Fig. 2c). However, except the gene *rpoA*, there were significant increases in the expression levels of PEP component genes, including *rpoB*, *rpoC1*, and *rpoC2*, in the *oscaf2* mutant relative to WT (Fig. 2c). By contrast, the expressions of photosynthesis-related genes involving *RbcS*, *RbcL*, *HEMA*, *CHLI*, *psbO*, *HEMD*, *psbA*, and *CHLM* were dramatically reduced in the *oscaf2* mutant (Fig. 2c). These results suggest that OsCAF2 might affect the expression level of chloroplast development and photosynthesis-related genes.

### Chloroplast development was defected in *oscaf2* mutant

To further study the albino leaf phenotype in the *oscaf2* mutant, we observed the ultrastructure of chloroplasts of leaves in WT and *oscaf2* in seedling stage using transmission electron microscopy (TEM). Our result showed that the integrated chloroplast structures and normal grana stacks were found in WT leaves (Fig. 2d-e), and the abnormal chloroplast structure and the disorganized grana lamellar stacks in *oscaf2* mutants (Fig. 2f-g). These data suggest that the mutation to *OsCAF2* impaired chloroplasts development in rice. These results also suggest that OsCAF2 is necessary for chloroplast development.

### OsCAF2 influences splicing of group II introns

**Fig. 3:**
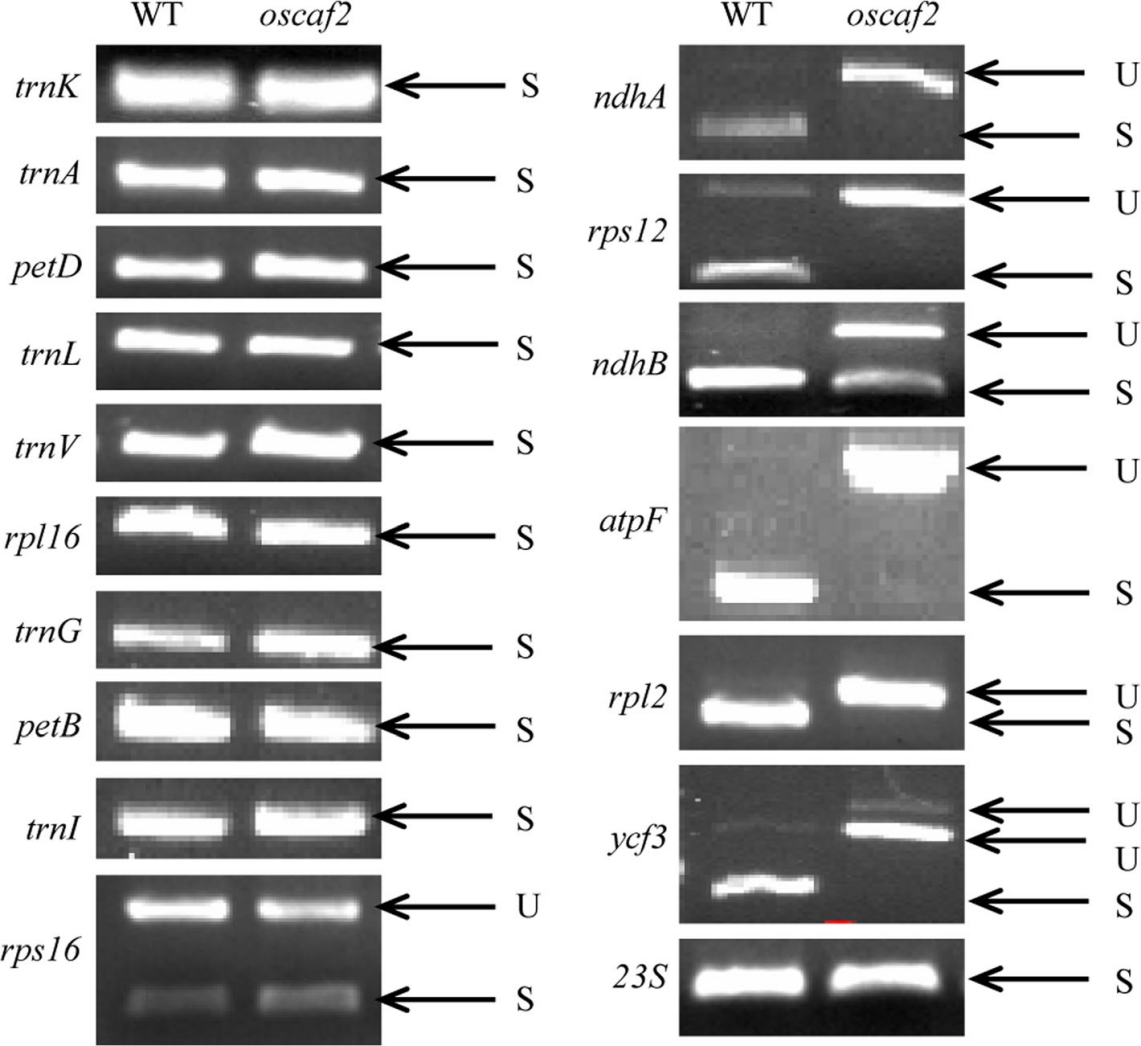
Splicing analysis of group I and group II introns in WT and *oscaf2* mutants. U indicates unspliced transcripts. S indicates spliced transcripts

In *A. thaliana* and maize, CAF2 promoted the splicing of the chloroplast RNA group IIB introns [25]. Therefore, we examined whether OsCAF2 was involved in chloroplast RNA intron splicing in rice. The rice chloroplast genome contains 17 group II introns and 1 group I intron. We amplified chloroplast genes that contained at least one intron using RT-PCR and compared intron splicing efficiency between the WT and *oscaf2* mutant. We found abnormal intron splicing of subgroup IIA introns (*rpl2*, *rps12*, and *atpF*) and subgroup IIB introns (*ndhA*, *ndhB*, and *ycf3*) in the *oscaf2* mutant, compared with the WTs (Fig. 3, Fig. S1)*.* However, the intron splicing of *trnL* (group I) was not affected in the *oscaf2* mutant (Fig. 3, Fig. S1). These results suggested that OsCAF2 might participate in splicing of subgroup IIA and subgroup IIB introns but not group I introns.

### Expression patterns and subcellular localization of OsCAF2

**Fig. 4:**
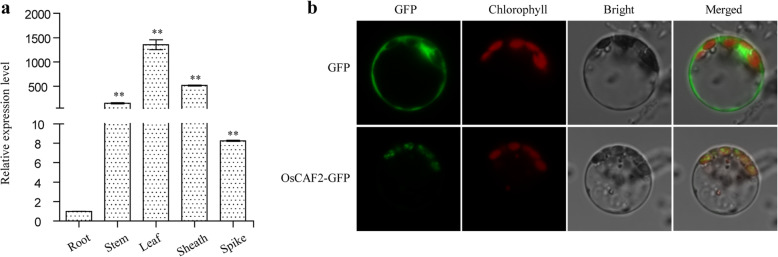
Expression pattern and subcellular localization of OsCAF2. **a** Expression pattern of OsCAF2 in various tissues, including root, stem, leaf, sheath, and spike at the heading stage. The values represent the mean of three independent experiments. Error bars indicate SE, ** indicates *P* < 0.01. **b** Subcellular localization of OsCAF2 protein in rice protoplasts

According to the gene expression and protein tools of rice (http://www.bar.utoronto.ca/efprice/cgi-bin/efpWeb.cgi), *OsCAF2* was highly expressed in leaves but low or undetectable level was found in spikes and root. To validate the result, we analyzed the expression pattern of *OsCAF2* in different tissues at the heading stage of *Nipponbare* using qRT-PCR. Compared with the root, the expression level of other tissues were significantly increased, and the gene expression in leaves even more than a thousand times (Fig. 4a). Our results indicated that *OsCAF2* expression was highest in green tissues, especially in leaves.

The Wolf PSORT (https://wolfpsort.hgc.jp/) website predicted that OsCAF2 was localized to rice chloroplasts. To determine the subcellular localization of OsCAF2, we constructed the OsCAF2-GFP fusion vector and transformed it into rice protoplasts. The p580 empty vector was used as a control. The results showed that the empty vector had green fluorescent signals in the nucleus, membrane, and cytoplasm, but the OsCAF2-GFP fusion protein produced signals that co-localized with the chloroplast autofluorescence signals, suggesting that OsCAF2 localizes in chloroplasts (Fig. 4b, Fig. S2). This result supports the view that OsCAF2 plays an important role in regulating chloroplast development.

### C-terminal region of OsCAF2 interacts with OsCRS2

**Fig. 5:**
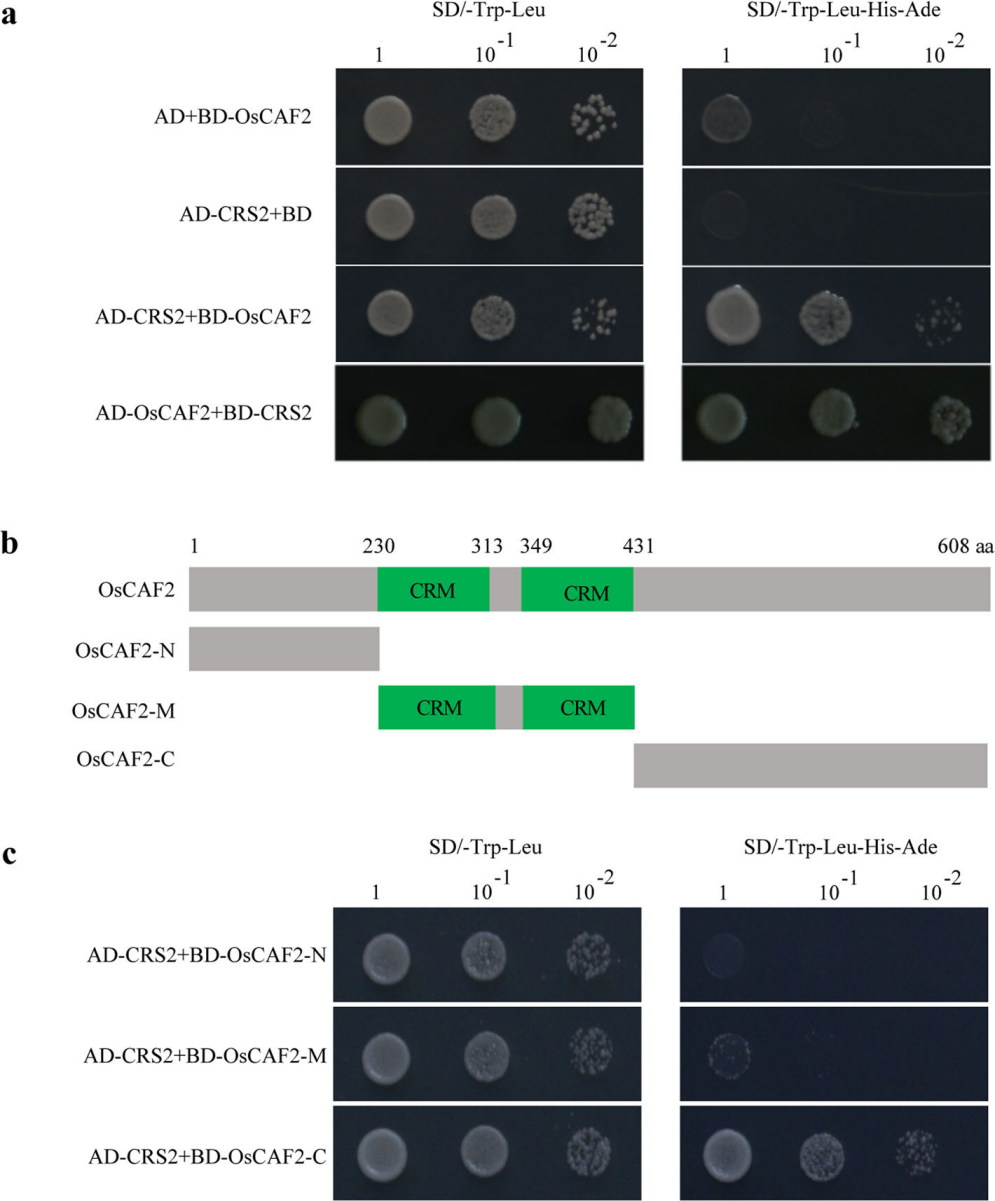
OsCAF2 interacts with OsCRS2 through the C-terminal region. **a** The Y2H interaction between OsCAF2 and OsCRS2. The full-length CDS of OsCAF2 and OsCRS2 were cloned into pGBKT7 (BD) and pGADT7 (AD) vectors to produce OsCAF2-BD, OsCAF2-AD, OsCRS2-BD, and OsCRS2-AD plasmids, respectively. The OsCAF2-BD and OsCRS2-AD, OsCAF2-AD and OsCRS2-BD were co-transformed into the AH109 yeast strain. The OsCAF2-BD and AD plasmid, and OsCRS2-AD and BD plasmid were co-transformed into the AH109 yeast strain as a control. **b** Schematic diagram of OsCAF2 protein structure. Different sections of OsCAF2 involving in OsCAF2-N (1–230 aa), OsCAF2-M (231–430 aa), and OsCAF2-C (431–608 aa). **c** Y2H interaction of OsCRS2 and different truncated sections of OsCAF2. The truncated sections of OsCAF2 were cloned into the BD vector to generate OsCAF2-N-BD, OsCAF2-M-BD, and OsCAF2-C-BD vectors. These three vectors were co-transferred into AH109 yeast cells with the OsCRS2-AD vector. The yeast cells were cultured on SD-T/L and SD−/T/L/H/A media with different dilution series (1, 10^− 1^, and 10^− 2^)

Previous studies show that the N-terminal region of CAF2 interacts with CRS2 and forms the CAF2-CRS2 complex in maize [23]. To confirm the relationship between CAF2 and CRS2 in rice, we acquired the coding sequence (CDS) of *OsCRS2* (*LOC_Os01g04130*) from rice leaves and constructed the OsCRS2-PGADT7 (OsCRS2-AD) and OsCAF2-PGBKT7 (OsCAF2-BD) vectors for yeast two-hybrid (Y2H) analysis. The AH109 yeast cell that contained either OsCRS2-AD and the empty BD vector or OsCAF2-BD and the empty AD vector were able to grow on synthetic dextrose medium lacking Leu and Trp (SD-T/L), but they were unable to grow on medium lacking Leu, Trp, His, and Ade (SD-L/T/H/A). However, the AH109 yeast cell that contained OsCRS2-AD and OsCAF2-BD, OsCRS2-BD and OsCAF2-AD were able to grow in SD-T/L and SD-L/T/H/A (Fig. 5a, Fig. S3). These results suggest that OsCAF2 can interact with OsCRS2 to form the OsCAF2-OsCRS2 complex in rice. We then truncated the OsCAF2 protein into three sections, named OsCAF2-N (N-terminal), OsCAF2-M (middle), and OsCAF2-C (C-terminal) and repeated the Y2H experiment (Fig. 5b). Among these sections, OsCAF2-M contains the two CRM domains. The OsCAF2-C interacted with OsCRS2 but OsCAF2-N and OsCAF2-M did not (Fig. 5c, Fig. S3). These results indicate that the C-terminal of OsCAF2 is necessary for the interaction with OsCRS2.

### OsCAF2 forms homodimers through the N-terminal region

**Fig. 6:**
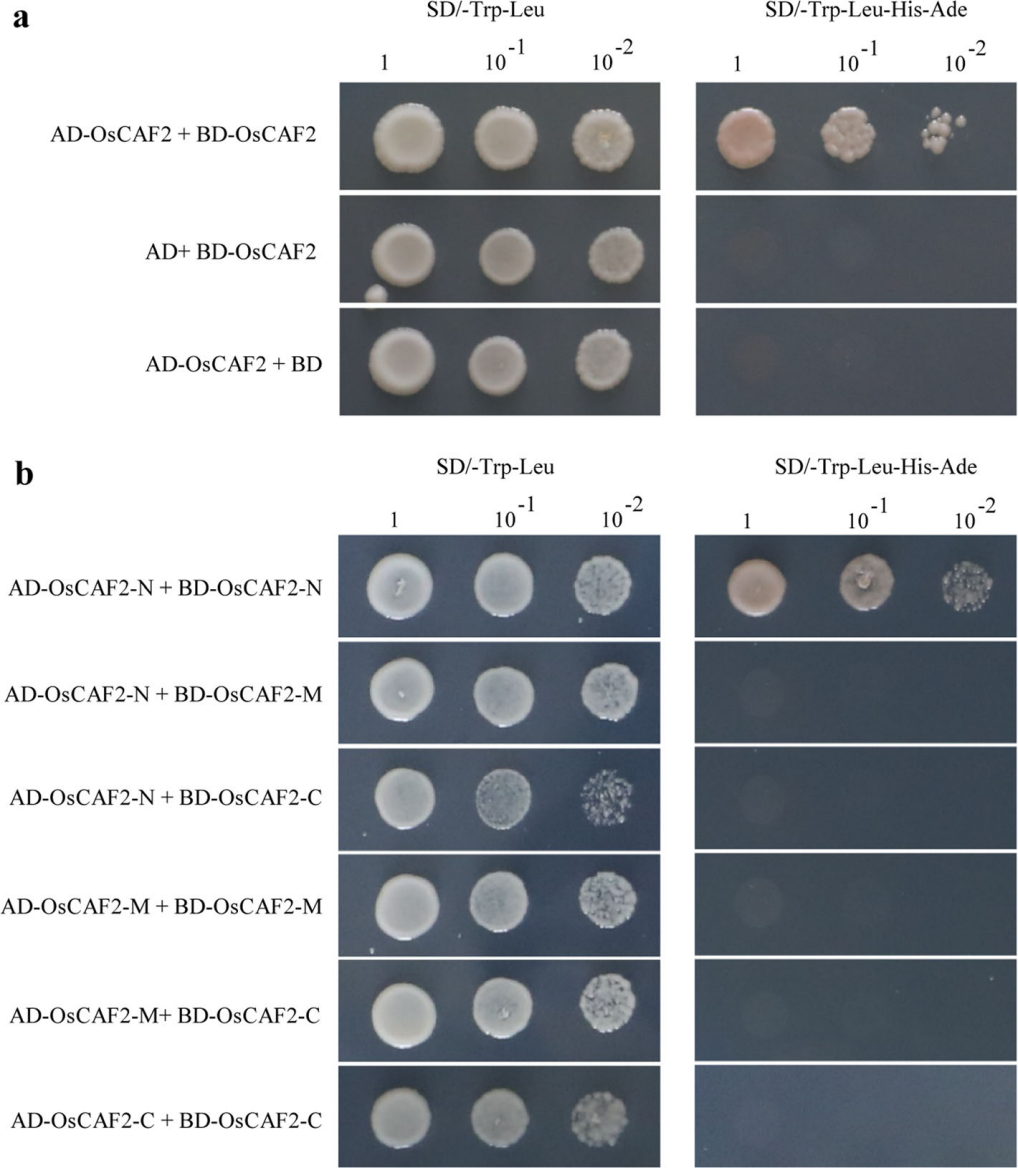
OsCAF2 interacts with itself through the N-terminal region. **a** Y2H interaction of OsCAF2 with itself. The full-length CDS of OsCAF2 was cloned into BD and AD vectors, to produce OsCAF2-BD and OsCAF2-AD vectors, respectively. The OsCAF2-BD and OsCAF2-AD were co-transformed into the AH109 yeast strain. The OsCAF2-BD and AD plasmid, and the OsCAF2-AD and BD plasmid were co-transformed into the AH109 yeast strain as control. **b** Y2H interaction of OsCAF2 with itself. The truncated sections of OsCAF2 were cloned into BD and AD vectors to produce the OsCAF2-N-BD, OsCAF2-M-BD, OsCAF2-C-BD, OsCAF2-N-AD, OsCAF2-M-AD and OsCAF2-C-AD vectors. A pair of AD and BD was co-transformed into the AH109 yeast strain. The yeast cells were cultured on SD-T/L and SD−/T/L/H/A media with different dilution series (1, 10^− 1^, and 10^− 2^)

In order to study whether OsCAF2 could form homodimers, we carried out an Y2H analyzes. In brief, we constructed OsCAF2-AD and OsCAF2-BD vectors and then co-transformed them into AH109 yeast cells. The Y2H results showed that OsCAF2 could self-interact and form homodimers (Fig. 6a, Fig. S4). We divided OsCAF2 into the three sections as above, to further analyze which section was responsible for forming homodimers. Only the N-terminal region of OsCAF2 was able to interact with itself, but it could not interact either with the M or C-terminal regions of OsCAF2 (Fig. 6b, Fig. S4). Therefore, the N-terminal of OsCAF2 was necessary to form homodimers.

## Discussion

The OsCAF2 protein has two CRM domains with homology to AtCAF2 and ZmCAF2. Our phenotypic results of the *oscaf2* mutant were consistent with the albino mutant *atcaf2* in *A. thaliana*, which was obtained by T-DNA insertion [25]. Therefore, we suspected that the function of CAF2 might be highly conserved in *A. thaliana* and rice. In rice, *AL2*, *OsCAF1*, and *CFM3* encoding proteins contained CRM domains, and the *al2*, *oscaf1*, and *oscfm3* mutants showed albino leaf phenotypes with disrupted chloroplast structure [11, 26, 27]. Research shows there were 14 proteins containing one or more CRM domains, and four of them contained two CRM domains in rice [7]. As the albino phenotypic of *oscaf2* mutants, we speculated that the function of OsCAF2 was not redundant with the other three proteins that contain two CRM domains. These results suggest that CRM domain proteins play a key role in chloroplast development in rice.

It has been reported that CAF2 is a splicing factor that is encoded by nuclear genes that participate in intron splicing of *petB*, *rps12*, *ndhA*, and *ycf3* in maize and *A. thaliana* [25]. This indicates that the molecular mechanism of CAF2 regulation of chloroplast development is relatively conserved in maize and *A. thaliana*. In this study, by analyzing the intron splicing of chloroplast genes, we found that OsCAF2 promotes the splicing of subgroup IIB and subgroup IIA introns. For example, OsCAF2 influences *rpl2* and *atpF* intron splicing, but ZmCAF2 and AtCAF2 does not influence splicing of these two introns in maize and *A. thaliana.* However, OsCAF2, AtCAF2, and ZmCAF2 all affect splicing of *ndhA* and *ycf3* introns. Therefore, this suggests that OsCAF2 has a conserved but distinct role compared to AtCAF2 and ZmCAF2. Similar to OsCAF1 and AL2 in rice, OsCAF2 affects splicing of subgroup IIA and subgroup IIB introns [11, 27]. The molecular mechanism of OsCAF2 regulating chloroplast development may be different from AtCAF2 and ZmCAF2. The function of OsCAF2 in splicing of subgroup IIA introns, such as *atpF* and *rpl2* introns, requires further study.

In plants, chloroplast development is mainly divided into three steps [28]. NEP and PEP participate in the second and third steps to regulate the transcription of plastid genes [28]. In the *oscaf2* mutant, the expression levels of the NEP component (*OsRpoTp*) are similar to the WT, while the expression of PEP components (*rpoB*, *rpoC1,* and *rpoC2*) increased. This might be caused by a feedback regulation mechanism: impaired chloroplast development might induce transcription of genes with PEP components. Similar conclusions were acquired in many chloroplast defective mutants, such as *asl3* and *sal4*, in rice. Previous work showed that *asl3* and *sal4* also exhibited albino leaf phenotypes and expression levels of *rpoB*, *rpoC1*, and *rpoC2* in *asl3* and *sal4* increased significantly [18, 29]. By contrast, expression levels of photosynthesis-related genes, such as *RbcS*, *RbcL*, *HEMA*, *CHLI*, *psbO*, *HEMD*, *psbA*, and *CHLM* decreased. It may be possible that the signals from the plasmid to the nucleus might be different in the *oscaf2* mutant. This result of signal transduction was also consistent with previous results in the *osptac2* mutant [30]. Thus, these results showed that OsCAF2 might influence chloroplast development through its effects on PEP and photosynthesis-related gene expression.

It was reported that CAF2 interacted with CRS2 through its C-terminal region in maize [23]. CRS2 was a group II intron splicing factor that could promote the splicing of nine group II introns [31, 32]. Gene expression analysis showed that expression levels of OsCAF2 were highest in leaves. The expression pattern of OsCAF2 was similar to OsCRS2. In this study, we determined that OsCAF2 interacts with OsCRS2 through its C-terminal region. Previous analysis of the C-terminal region of CAF2 in monocot and dicot plants, including *Arabidopsis thaliana*, maize and rice, found that there was a conservative residue of CAF2 that formed a hydrophobic region necessary for protein interactions with CRS2 [23]. In rice, OsCAF2 might interact with OsCRS2 through this conservative residue. The molecular mechanism of the CAF2-CRS2 complex formation might be conserved across plants. It has been reported that the homodimers and heterodimers might have biological advantages than the individual protein, which could promote the synthesis of products or regulate the activity of partner enzyme domains [33]. Consider the homodimers of OsCAF2 formed via its N-terminal region, we suspected that proper chloroplast development requires recruitment of OsCAF2 and OsCRS2 to form the OsCAF2-OsCRS2 complex, as well homodimers of OsCAF2 to achieve splicing of group II introns. Previous studies have shown that OsCAF1 also contains two CRM domains that could affect splicing of subgroup IIA and subgroup IIB introns, and the orthologous genes of AtCAF1 and ZmCAF1 in *A. thaliana* and *maize* only promoted the splicing of subgroup IIB introns [27]. In addition, OsCAF1 interacts with itself to form homodimers. In maize, ZmCRS1 contains three CRM domains and can form homodimers, but whether CAF2 forms homodimers has not been reported in maize and *A. thaliana* [22]. Therefore, this study provides new clues to the function of the CAF2 protein in different plants and provides important information for exploring the development of chloroplasts in rice.

## Conclusions

The present study generated albino lethal *oscaf2* mutants using CRISPR/Cas9 gene-editing system. The following study showed that *OsCAF2* could affect chloroplast development, and might participate in the splicing of group IIA and IIB introns, which differs from its orthologs in *A. thaliana* and maize. The Y2H assay showed that the C-terminal region of OsCAF2 interacted with OsCRS2 and formed an OsCAF2-OsCRS2 complex, and the N-terminal region of OsCAF2 interacted with itself to form homodimers. These findings improved our understanding of the OsCAF2 protein, and revealed additional information about the molecular mechanism of OsCAF2 in regulating of chloroplast development in rice.

## Methods

### Plant materials and growth conditions

The japonica variety *Nipponbare* have been conventionally cultivated in Japan. The seeds of *Nipponbare* were provided by the China National Rice Research Institute (CNRRI) in Hangzhou, Zhejiang Province, China. We designed the two target sites of *OsCAF2* (GTGCGGCTGCACACCCCGCTCGG, AAGGTTATTCACCGAGTAGGTGG) and constructed the pC1300-*Ubi*::Cas9-gX^*OsCAF2-g1*^-gX^*OsCAF2-g2*^ vector, according to previously-described method [34]. Vector construction and sequencing related primers are listed in Table S1. This vector were transformed into *Nipponbare* via *Agrobacterium*-mediated transformation. The *OsCAF2* mutation of the positive transgenic plants were identified by the Sanger method and analyzed by the degenerate sequence decoding method [35]. All the transgenic plants we obtained and their offspring were grown in the greenhouse under constant temperature (30 °C at 16 h light and 8 h dark) of CNRRI in Hangzhou, Zhejiang Province, China.

### Pigment content measurement

The Chlorophyll a (Chla), chlorophyll b (Chlb), carotenoid (Car) and total chlorophyll (Chl) content was measured. In brief, we obtained WT and *oscaf2* mutant leaves (0.2 g) at the seedling stage. The leaves were cut and soaked in 5 ml 95% ethanol for 48 h under dark conditions. Spectrophotometric measurements were conducted using a UV-1800PC spectrophotometer (Mapada, China) at 470 nm, 649 nm, and 665 nm. According to previous methods [36], we calculated the Chla, Chlb, Car and Chl content in WT and *oscaf2* mutant leaves.

### Transmission electron microscopy (TEM) assays

At the three leaf stage, we samples of WT and *oscaf2* mutant leaves were cut into 0.1 cm × 0.1 cm pieces. The samples were fixed in 2.5% glutaraldehyde for 4 h in at 4 °C (pH 7.0) and then fixed in 1% OsO4 (pH 7.4). The fixed samples were stained with uranyl acetate. After dehydration in an ethanol series, the samples were embedded in acrylic resin. The samples were staining and observed with a Hitachi-7500 (Tokyo, Japan) transmission electron microscope.

### RNA extraction and quantitative real-time PCR (qRT-PCR)

Total RNA was extracted from leaves using RNA extraction kit (Axygen, USA) according to the manufacturer’s protocols. First-strand cDNA was synthesized using the ReverTra Ace qPCR RT Kit (TOYOBO, Japan) according to the manufacturer’s instructions. The qRT-PCR analysis process was as follows: 5 min at 94 °C followed by 40 cycles of 95 °C for 15 s and 58 °C for 50 s. The *OsActin1* was used as a reference gene in this study. The relative expression levels were calculated according to the 2^−ΔΔCT^ method [37]. All primers for qRT-PCR are shown in Table S1.

### Splicing analysis of group I and group II introns

We used RT-PCR to perform splicing analysis of group I and group II introns of chloroplast genes from WT and *oscaf2* mutants. The RT-PCR procedure was as follows: 94 °C for 5 min, followed by 30 cycles of 94 °C for 30 s, 58 °C for 30 s, 72 °C for 45 s, and a final elongation step at 72 °C for 10 min. All chloroplast RNA splicing analysis primers are listed in Table S1.

### Subcellular localization of OsCAF2

To confirm subcellular localization of the OsCAF2 protein, the full-length *OsCAF2* coding sequence (remove stop code) was amplified and cloned into the pAN580 vector to create the p580–2 × CaMV35S::OsCAF2-GFP (*OsCAF2-GFP*) plasmid (Table S2). The *OsCAF2-GFP* vector construction primers are listed in Table S1. The preparation of rice protoplasts and transformation followed previously described methods [38]. The fluorescence of green fluorescent protein (GFP) was observed using confocal laser scanning microscopy (Zeiss LSM 780, Germany).

### Yeast two-hybrid (Y2H) assays

The full-length and truncated coding sequences of *OsCAF2*, and full-length *OsCRS2* coding sequence were amplified via PCR (Table S2). Relevant PCR amplification primers are listed in Table S1. PCR amplification products were cloned into the pGAD-T7 or pGBK-T7 vectors. The Y2H experiment used the Clontech two-hybrid system, according to the manufacturer’s instructions.

## Supplementary information

**Additional file 1: Table S1.** The primers used in present study. **Table S2.** Sequence of *OsCAF2* and *OsCRS2* genes from *Nipponbare.*

**Additional file 2: Figure S1.** Original images for Fig. 3.

**Additional file 3: Figure S2.** Original images for Fig. 4b.

**Additional file 4: Figure S3.** Original images for Fig. 5.

**Additional file 5: Figure S4.** Original images for Fig. 6.

## Data Availability

The datasets supporting the results of this article are available from the corresponding author on reasonable request. Sequence data used during the current study for the cDNA and genomic DNA of *OsCAF2* and *OsCRS2* are available from the GenBank/EMBL data libraries under accession numbers *LOC_Os01g21990*, *LOC_Os01g04130*, respectively, and could also available from the National Center for Biotechnology Information (NCBI) (OsCAF2 Gene ID: 4326994, OsCRS2 Gene ID: 4325801).

## Abbreviations

PEPs: Plastid-encoded polymerases
NEPs: Nucleus-encoded polymerases
pre-mRNA: Precursor messenger mRNA
PPR: Pentatricopeptide repeat
CRM: Chloroplast RNA splicing and ribosome maturation
CRISPR: Clustered regularly interspaced short palindromic repeats
TEM: Transmission electron microscopy
Y2H: Yeast two-hybrid
qRT-PCR: RNA extraction and Quantitative real-time PCR
GFP: Green fluorescent protein
CDS: Coding sequence

## References

[1] Jarvis P, López-Juez E (2013). Biogenesis and homeostasis of chloroplasts and other plastids. Nat Rev Mol Cell Biol.

[2] Li Y, Zhang J, Li L, Gao L, Xu J, Yang M (2018). Structural and comparative analysis of the complete chloroplast genome of *Pyrus hopeiensis*- “wild plants with a tiny population” -and three other *Pyrus* species. Int J Mol Sci.

[3] Bobik K, Burch-Smith TM (2015). Chloroplast signaling within, between and beyond cells. Front Plant Sci.

[4] Nakai M (2018). New perspectives on chloroplast protein import. Plant Cell Physiol.

[5] Hedtke B, Börner T, Weihe A (1997). Mitochondrial and chloroplast phage-type RNA polymerases in Arabidopsis. Science..

[6] Yu QB, Huang C, Yang ZN (2014). Nuclear-encoded factors associated with the chloroplast transcription machinery of higher plants. Front Plant Sci.

[7] De Longevialle AF, Small ID, Lurin C (2010). Nuclearly encoded splicing factors implicated in RNA splicing in higher plant organelles. Mol Plant.

[8] Bonen L, Vogel J (2001). The ins and outs of group II introns. Trends Genet.

[9] Till B, Schmitz-Linneweber C, Williams-Carrier R, Barkan A (2001). CRS1 is a novel group II intron splicing factor that was derived from a domain of ancient origin. Rna..

[10] Barkan A, Goldschmidt-Clermont M (2000). Participation of nuclear genes in chloroplast gene expression. Biochimie..

[11] Liu C, Zhu H, Xing Y, Tan J, Chen X, Zhang J (2016). *Albino Leaf 2* is involved in the splicing of chloroplast group I and II introns in rice. J Exp Bot.

[12] Khrouchtchova A, Monde RA, Barkan A (2012). A short PPR protein required for the splicing of specific group II introns in angiosperm chloroplasts. Rna..

[13] Rovira AG, Smith AG (2019). PPR proteins - orchestrators of organelle RNA metabolism. Physiol Plant.

[14] Schmitz-Linneweber C, Williams-Carrier RE, Williams-Voelker PM, Kroeger TS, Vichas A, Barkan A (2006). A pentatricopeptide repeat protein facilitates the trans-splicing of the maize chloroplast rps12 pre-mRNA. Plant Cell.

[15] Tadini L, Ferrari R, Lehniger MK, Mizzotti C, Moratti F, Resentini F (2018). Trans-splicing of plastid rps12 transcripts, mediated by AtPPR4, is essential for embryo patterning in *Arabidopsis thaliana*. Planta..

[16] De Longevialle AF, Hendrickson L, Taylor NL, Delannoy E, Lurin C, Badger M (2008). The pentatricopeptide repeat gene *OTP51* with two LAGLIDADG motifs is required for the *cis*-splicing of plastid *ycf3* intron 2 in *Arabidopsis thaliana*. Plant J.

[17] Chateigner-Boutin AL, Des Francs-Small CC, Delannoy E, Kahlau S, Tanz SK, De Longevialle AF (2011). OTP70 is a pentatricopeptide repeat protein of the E subgroup involved in splicing of the plastid transcript *rpoC1*. Plant J.

[18] Wang Z, Lv J, Xie S, Zhang Y, Qiu Z, Chen P (2018). *OsSLA4* encodes a pentatricopeptide repeat protein essential for early chloroplast development and seedling growth in rice. Plant Growth Regul.

[19] Chen L, Huang L, Dai L, Gao Y, Zou W, Lu X (2019). *PALE-GREEN LEAF12* encodes a novel Pentatricopeptide repeat protein required for chloroplast development and 16S rRNA processing in Rice. Plant Cell Physiol.

[20] Beick S, Schmitz-Linneweber C, Williams-Carrier R, Jensen B, Barkan A (2008). The pentatricopeptide repeat protein PPR5 stabilizes a specific tRNA precursor in maize chloroplasts. Mol Cell Biol.

[21] Wang Y, Ren Y, Zhou K, Liu L, Wang J, Xu Y (2017). *WHITE STRIPE LEAF4* encodes a novel P-type PPR protein required for chloroplast biogenesis during early LEAF development. Front Plant Sci.

[22] Ostersetzer O, Cooke AM, Watkins KP, Barkan A (2005). CRS1, a chloroplast group II intron splicing factor, promotes intron folding through specific interactions with two intron domains. Plant Cell.

[23] Ostheimer GJ, Rojas M, Hadjivassiliou H, Barkan A (2006). Formation of the CRS2-CAF2 group II intron splicing complex is mediated by a 22-amino acid motif in the COOH-terminal region of CAF2. J Biol Chem.

[24] Ostheimer GJ, Williams-Carrier R, Belcher S, Osborne E, Gierke J, Barkan A (2003). Group II intron splicing factors derived by diversification of an ancient RNA-binding domain. EMBO J.

[25] Asakura Y, Barkan A (2006). Arabidopsis orthologs of maize chloroplast splicing factors promote splicing of orthologous and species-specific group II introns. Plant Physiol.

[26] Asakura Y, Bayraktar OA, Barkan A (2008). Two CRM protein subfamilies cooperate in the splicing of group IIB introns in chloroplasts. Rna..

[27] Zhang Q, Shen L, Wang Z, Hu G, Ren D, Hu J (2019). OsCAF1, a CRM domain containing protein, influences chloroplast development. Int J Mol Sci.

[28] Zhang Z, Cui X, Wang Y, Wu J, Gu X, Lu T (2017). The RNA editing factor WSP1 is essential for chloroplast development in Rice. Mol Plant.

[29] Lin D, Gong X, Jiang Q, Zheng K, Zhou H, Xu J (2015). The rice *ALS3* encoding a novel pentatricopeptide repeat protein is required for chloroplast development and seedling growth. Rice (New York, NY).

[30] Wang D, Liu H, Zhai G, Wang L, Shao J, Tao Y (2016). *OspTAC2* encodes a pentatricopeptide repeat protein and regulates rice chloroplast development. J Genet Genomics.

[31] Jenkins BD, Kulhanek DJ, Barkan A (1997). Nuclear mutations that block group II RNA splicing in maize chloroplasts reveal several intron classes with distinct requirements for splicing factors. Plant Cell.

[32] Jenkins BD, Barkan A (2001). Recruitment of a peptidyl-tRNA hydrolase as a facilitator of group II intron splicing in chloroplasts. EMBO J.

[33] Panicot M, Minguet EG, Ferrando A, Alcázar R, Blázquez MA, Carbonell J (2002). A polyamine metabolon involving aminopropyl transferase complexes in Arabidopsis. Plant Cell.

[34] Wang C, Shen L, Fu Y, Yan C, Wang K (2015). A simple CRISPR/Cas9 system for multiplex genome editing in Rice. J Genet Genom.

[35] Ma X, Chen L, Zhu Q, Chen Y, Liu Y (2015). Rapid decoding of sequence-specific nuclease-induced heterozygous and Biallelic mutations by direct sequencing of PCR products. Mol Plant.

[36] Lichtenthaler H (1987). Chlorophylls and carotenoids: pigments of photosynthetic biomembranes. Methods Enzymol.

[37] Livak KJ, Schmittgen TD (2001). Analysis of relative gene expression data using real-time quantitative PCR and the 2^−ΔΔCT^ method. Methods..

[38] Zhang Y, Su J, Duan S, Ao Y, Dai J, Liu J (2011). A highly efficient rice green tissue protoplast system for transient gene expression and studying light/chloroplast-related processes. Plant Methods.

