# Supplementary material for: OsCAF2 contains two CRM domains and is necessary for chloroplast development in rice
Source: BMC Plant Biol. 2020 Aug 18;20:381. doi: 10.1186/s12870-020-02593-z (PMC7437035; doi:10.1186/s12870-020-02593-z)
Supplement: Supplementary file 1 — Additional file 1: Table S1. The primers used in present study. Table S2. Sequence of OsCAF2 and OsCRS2 genes from Nipponbare. [file 12870_2020_2593_MOESM1_ESM.docx]

Table S1. The primers used in present study

| Primer name | Sequence (5’-3’) | Experiment |
| --- | --- | --- |
| OsCAF2-g1++ | GGCAGTGCGGCTGCACACCCCGCT | Vector construction |
| OsCAF2-g1-- | AAACAGCGGGGTGTGCAGCCGCAC | Vector construction |
| OsCAF2-g2++ | GGCAAAGGTTATTCACCGAGTAGG | Vector construction |
| OsCAF2-g2-- | AAACCCTACTCGGTGAATAACCTT | Vector construction |
| OsCAF2-jc-F1 | TCGACTTCCGGTTCAGCTAC | Detection of target mutations |
| OsCAF2-jc-R1 | CCATGTTGCTACCCAATGTG | Detection of target mutations |
| OsCAF2-jc-F2 | GAGCCTGGACTGGAGGACTT | Detection of target mutations |
| OsCAF2-jc-R2 | AAGGGGTTCTGAACATCGTG | Detection of target mutations |
| rbcL-F | CTTGGCAGCATTCCGAGTAA | qRT-PCR assay |
| rbcL-R | ACAACGGGCTCGATGTGATA | qRT-PCR assay |
| PsbA-F | ACTAGCACCGAAAACCGTCTTT | qRT-PCR assay |
| PsbA-R | CAGCGATGAAGGCGATAATAAA | qRT-PCR assay |
| OsActin-F | CGGGAAATTGTGAGGGACATG | qRT-PCR assay |
| OsActin-R | AGGAAGGCTGGAAGAGGACC | qRT-PCR assay |
| RbcS-F | GTGGCAACTAAGCCGTCATCGTC | qRT-PCR assay |
| RbcS-R | TGCCTCACCCAACAACATATAGTCGT | qRT-PCR assay |
| PsbO-F | TCGAGGAGAAGGACGGAATCGAC | qRT-PCR assay |
| PsbO-R | CTTTGGGTCGAGGAAGGACGAAC | qRT-PCR assay |
| HEMA-F | GAACTCACCAGTCTGAATCATATTGA | qRT-PCR assay |
| HEMA-R | CATCCAGTCTACCACTTCTCTAATCC | qRT-PCR assay |
| CHLM-F | CCATCCATTGGTCTCCTTATGACA | qRT-PCR assay |
| CHLM-R | GTAGCCTACTTACCATCAATGAGTC | qRT-PCR assay |
| CHLI-F | GTTCGAGCCTGGTTTGCTTGC | qRT-PCR assay |
| CHLI-R | CTCTCCACGGTGTTCCATCCTG | qRT-PCR assay |
| HEMD-F | TGGAAGGCTGCTGGAAATCCTAAG | qRT-PCR assay |
| HEMD-R | TCCTTGGAAGCTCTGAGGCCAA | qRT-PCR assay |
| PORA-F | TGTACTGGAGCTGGAACAACAACT | qRT-PCR assay |
| PORA-R | TCAATAGCACATCACTCTCACTCACT | qRT-PCR assay |
| rpoA-F | TGGTATTGCCTTGCGAAGAG | qRT-PCR assay |
| rpoA-R | TGTACCGATTCTTCAATACCTGC | qRT-PCR assay |
| YGL1-F | TGGACAGTTGAAGATGTT | qRT-PCR assay |
| YGL1-R | GAATAGGACGGTAAGGTT | qRT-PCR assay |
| RpoTp-F | TCCTCATGTCGAGCAAGGAT | qRT-PCR assay |
| RpoTp-R | TGAAAGAATGTCTGGACTTTGAAC | qRT-PCR assay |
| rpoC1-F | ATTAGACGCATGCAATTGGC | qRT-PCR assay |
| rpoC1-R | CAATGGGTCTTAATTCGGGAG | qRT-PCR assay |
| rpoC2-F | CAATTTACGCGAGGGACTTTCT | qRT-PCR assay |
| rpoC2-R | TGTAAGATATCCAGCATCCGCC | qRT-PCR assay |
| rpoB-F | TTTGGTTTCGATGTGCAAAAGC | qRT-PCR assay |
| rpoB-R | TATGGTCTAATTCCGAGCGGT | qRT-PCR assay |
| OsActin-F1 | CGGGAAATTGTGAGGGACATG | qRT-PCR assay |
| OsActin-R1 | AGGAAGGCTGGAAGAGGACC | qRT-PCR assay |
| OsCAF2-F | ACTACAACCCTCGAACCCG | qRT-PCR assay |
| OsCAF2-R | AAATTGGCAGCAGATCCTT | qRT-PCR assay |
| petB-F | TTCTCATATACGGTTCTCGG | Splicing analysis |
| petB-R | TAAAGGGCCCGAAATACCTT | Splicing analysis |
| rps12-F | ACTATCAACCCCAAAAAACC | Splicing analysis |
| rps12-R | TTTGGCTTTTTGACCCCAT | Splicing analysis |
| petD-F | ATGGGAGTAACAAAGAAACC | Splicing analysis |
| petD-R | TGTTGCTCCAATACCTAACC | Splicing analysis |
| rpl2-F | ACGGCGAAACATTTATACAA | Splicing analysis |
| rpl2-R | TTACTTACGGCGACGAAGAATA | Splicing analysis |
| rpl16-F | ATGCTTAGTCCCAAAAGAAC | Splicing analysis |
| rpl16-R | AACCGAAGAAATTGACTTCG | Splicing analysis |
| atpF-F | TTTTAGCTCACTGGCCATCC | Splicing analysis |
| atpF-R | TTCATCGCCCTTTGTTTTTC | Splicing analysis |
| ndhA-F | ATGATAATAGACAGGGTACAGG | Splicing analysis |
| ndhA-R | TTATAGTGAAACAAGTTGGGAAG | Splicing analysis |
| ndhB-F | ATGATCTGGCATGTACAGAATG | Splicing analysis |
| ndhB-R | CTAAAAGAGGGTATCCTGAGCA | Splicing analysis |
| rps16-F | AAAACGATGTGGTAGAAAGC | Splicing analysis |
| rps16-R | AGAATTCCGCCTTCCTTAAA | Splicing analysis |
| trnA-F | GGGGATATAGCTCAGTTGGT | Splicing analysis |
| trnA-R | TGGAGATAAGCGGACTCGAA | Splicing analysis |
| trnG-F | TCGTTAGCTTGGAAGGCTAG | Splicing analysis |
| trnG-R | GCGGGTATAGTTTAGTGGTA | Splicing analysis |
| trnI-F | TGGGCCATCCTGGACTTGA | Splicing analysis |
| trnI-R | AGCTCAGTGGTAGAGCGCG | Splicing analysis |
| ycf3-F | ATGCCTAGATCCCGTATAAATG | Splicing analysis |
| ycf3-R | TTATTCAAATTCAAAGCGCTTC | Splicing analysis |
| trnK-F | GGTTGCCCGGGACTCGAA | Splicing analysis |
| trnK-R | GGGTTGCTAACTCAATGGTAGAG | Splicing analysis |
| trnL-F | GGATATGGCGAAATCGGTA | Splicing analysis |
| trnL-R | TGGGGATAGAGGGACTTGA | Splicing analysis |
| trnV-F | TAGGGCTATACGGATTCGAA | Splicing analysis |
| trnV-R | AGGGCTATAGCTCAGTTCGG | Splicing analysis |
| 23S-F | TTCAAAAGAGGAAAGGCTTG | Splicing analysis |
| 23S-R | AGAGAGCACTCATCTTGGGG | Splicing analysis |
| P580-OsCAF2-F | GACAGCCCAGATCAACTAGTATGTCGCCGCCGCCGCCGCAGC | Subcellular localization assay |
| P580-OsCAF2-R | CCCTTGCTCACCATGGATCCCTCATCATTCAACTGTTTTACCACTGC | Subcellular localization assay |
| OsCAF2-AD-F | GGAGGCCAGTGAATTCATGTCGCCGCCGCCGCCGCAGC | Y2H assays |
| OsCAF2-AD-R | CGAGCTCGATGGATCCCTCATCATTCAACTGTTTTACCACTGC | Y2H assays |
| OsCAF2-BD-F | CATGGAGGCCGAATTCATGTCGCCGCCGCCGCCGCAGC | Y2H assays |
| OsCAF2-BD-R | GCAGGTCGACGGATCCCTCATCATTCAACTGTTTTACCACTGC | Y2H assays |
| OsCAF2-N-AD-F | GGAGGCCAGTGAATTCATGTCGCCGCCGCCGCCGCAGCGGC | Y2H assays |
| OsCAF2-N-AD-R | CGAGCTCGATGGATCCGAGCGGCTCGCCGAGCACCTCCTCC | Y2H assays |
| OsCAF2-M-AD-F | GGAGGCCAGTGAATTCACCGCCGCCGAGGTGCGCGACCTCG | Y2H assays |
| OsCAF2-M-AD-R | CGAGCTCGATGGATCCTCTGAACATTAATATCTGCTCATTG | Y2H assays |
| OsCAF2-C-AD-F | GGAGGCCAGTGAATTCGGCAAGGAATGGAAGTCCAGATATC | Y2H assays |
| OsCAF2-C-AD-R | CGAGCTCGATGGATCCTCACTCATCATTCAACTGTTTTACC | Y2H assays |
| OsCRS2-AD-F | GGAGGCCAGTGAATTCATGTCGCTTGCCGTGGCCACTCCCG | Y2H assays |
| OsCRS2-AD-R | CGAGCTCGATGGATCCTCAAACACTGTGGAACTTGTACTTC | Y2H assays |
| OsCRS2-BD-F | CATGGAGGCCGAATTCATGTCGCTTGCCGTGGCCACTCCCG | Y2H assays |
| OsCRS2-BD-R | GCAGGTCGACGGATCCTCAAACACTGTGGAACTTGTACTTC | Y2H assays |
| OsCAF2-C-BD-F | CATGGAGGCCGAATTCGGCAAGGAATGGAAGTCCAGATATC | Y2H assays |
| OsCAF2-C-BD-R | GCAGGTCGACGGATCCTCACTCATCATTCAACTGTTTTACC | Y2H assays |
| OsCAF2-M-BD-F | CATGGAGGCCGAATTCACCGCCGCCGAGGTGCGCGACCTCG | Y2H assays |
| OsCAF2-M-BD-R | GCAGGTCGACGGATCCTCTGAACATTAATATCTGCTCATTG | Y2H assays |
| OsCAF2-N-BD-F | CATGGAGGCCGAATTCATGTCGCCGCCGCCGCCGCAGCGGC | Y2H assays |
| OsCAF2-N-BD-R | GCAGGTCGACGGATCCGAGCGGCTCGCCGAGCACCTCCTCC | Y2H assays |

Table S2 Sequence of *OsCAF2* and *OsCRS2* genes from *Nipponbare*

| Gene | cDNA sequence |
| --- | --- |
| *OsCAF2* | atgtcgccgccgccgccgcagcggccatccccctcccgcgcaggccgcgccaacctcttctcgtccccgccgcctcccctccccaactgctacgaccccaagcaccgccgccccgcgccgccgcctctcccctccgcccgtcgcctccccagcaaccgccgccgccgccacgaccagcctccgaatccgaccaccggcaatggcgggaaccccgccttccgcgcgccgcacctccgcaccgcctaccgcaagcccgtcccacccgtggcggcggcgggggagggcgaggcgctcctcgccgccgacgccagcgacgccgccgacgggcgggccgtggtggtggggccgtcggggctgtcgttccggctcccgggcgcgccgttcgacttccggttcagctactccgagtgcccccgcgccccgccggtggccatccgggagcccgcgttcctgccgttcgcgccgccgacgatgccccggccgtggacggggaaggcgccgctgctgaccaaggaggagaaggcgcggcggcggggcgtgcggctgcacaccccgctcggggaggaggcgcccaggacggtgagcgcgcacgggatcatgatggaggtgagggggaggaggaagctggacctggccagggtcagccccggcgacggcaggagcagggaggaggtgctcggcgagccgctcaccgccgccgaggtgcgcgacctcgtcaagccccacatctcgcacaaccgccagctcaacatcggaagggatggattgacccacaacatgttggaaatgatacattgtcattggaggcgccaggaaatttgcaaagtgagatgccgaggagttccaactgttgatatgaagaatctgtgctatcaccttgaggaaaaatcaggcggaaaggttattcaccgagtaggtggtgtagttttcttataccgtggaagaaactacaaccctcgaacccggcctcgttatccactaatgctttggaagccagctactcctgtctatccaaaacttatacaggaagccccagaaggtcttacaaaagaggaagcagatgaaatgagaagaagagggaaggatctgctgccaatttgcaaattagcaaaaaatggtatatacatctatcttgtcagggatgttcgagatgcttttgagggaagtgatctggtgaagattgactgcgaggggttgaatccaagtgactacaaaaagattggagcaaaactaagggatcttgttccttgtgttcttttgtcgtttgacaatgagcagatattaatgttcagaggcaaggaatggaagtccagatatccaaagcctctgactcttattccaaaaattcgaaagaataatgttccaatgtcatctgatgagagtagttcagatgaagccactgatgatgatgaccgtttggcagtaagagaagtactgagacctaaaatgtttgaactatggacaaatgcgattgagtcatctgtggcactgatgttggatgatgctgaagtggatgctcttacacctgattcccttcttacaagggtagaggacttcagtgttacatctcaggttgtcgaacattcatttccagctgtgcttgtggccaatgatgaatcaaacccagatgtcctaaatgctgaatacaccgaagatgagcctgagacaggtacacttgaaccccaacaacatgagtttacagaatcttctgatgtcgcagaggatgatcatttcgaagatgacatgcttaagcgtttggagtcatcagtcccattgggagcattaccgatcgacgcagtggtaaaacagttgaatgatgagtga |
| *OsCRS2* | atgtcgcttgccgtggccactcccgcctccgcccgcctctccccgctcaccacctcctcccccgaaccctgccgccgccgccgcctcctcctctccgcagccgcccctctccggcgcacccgtctccggcgccgcatcgccgtcgtcgcgtccgtccccgaccccgcggcgcggccggcggagtacacgccctggctcatcgccggcctcggcaaccccggcagcaagtaccacggcacgcgtcacaacgtggggtttgagatggtcgatcgcatcgcgcgagacgaggggatcaccatgaacacgatccagtccaagtcgctgctcggaataggttcgattggggaagtgcctgtcttgttggtaaagccacaatcatatatcaactacagtggggaggcaattggacctcttgctgcctattatcaagtaccattgcggcatatccttgtgatgtatgatgagatgagtctacccaatggtgtattgcgtcttcaacggaaaggtggtcatggtcgccataatggcctgcagaacgtaatggaatgtttggacagttcccgtgaacttcctcgtttatctataggcattggtagcccacccggcaaaatggacacgcgtgcttttcttctgcagaagttcagttcagaggaacggctgcagattgacactgctttggagcaaggggtggatgctgtgagaactcttgtgttgaagggttttagtggaagtattgagagattcaatctggttcagaagtacaagttccacagtgtttga |
