# Supplementary material for: OsCAF2 contains two CRM domains and is necessary for chloroplast development in rice
Source: BMC Plant Biol. 2020 Aug 18;20:381. doi: 10.1186/s12870-020-02593-z (PMC7437035; doi:10.1186/s12870-020-02593-z)
Supplement: Supplementary file 2 — Additional file 2: Figure S1. Original images for Fig. 3. [file 12870_2020_2593_MOESM2_ESM.docx]

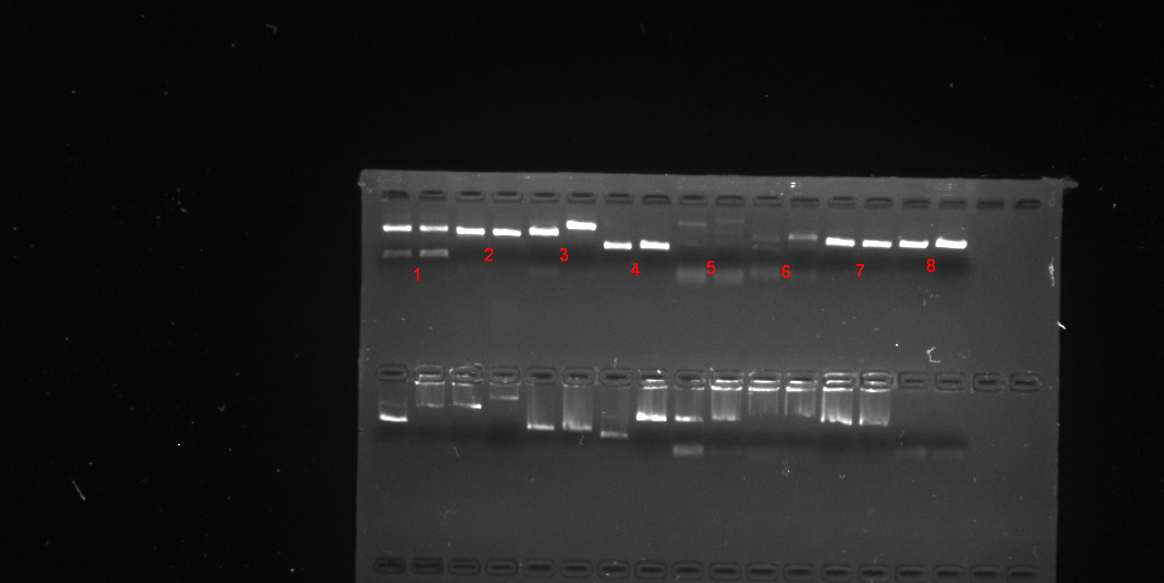

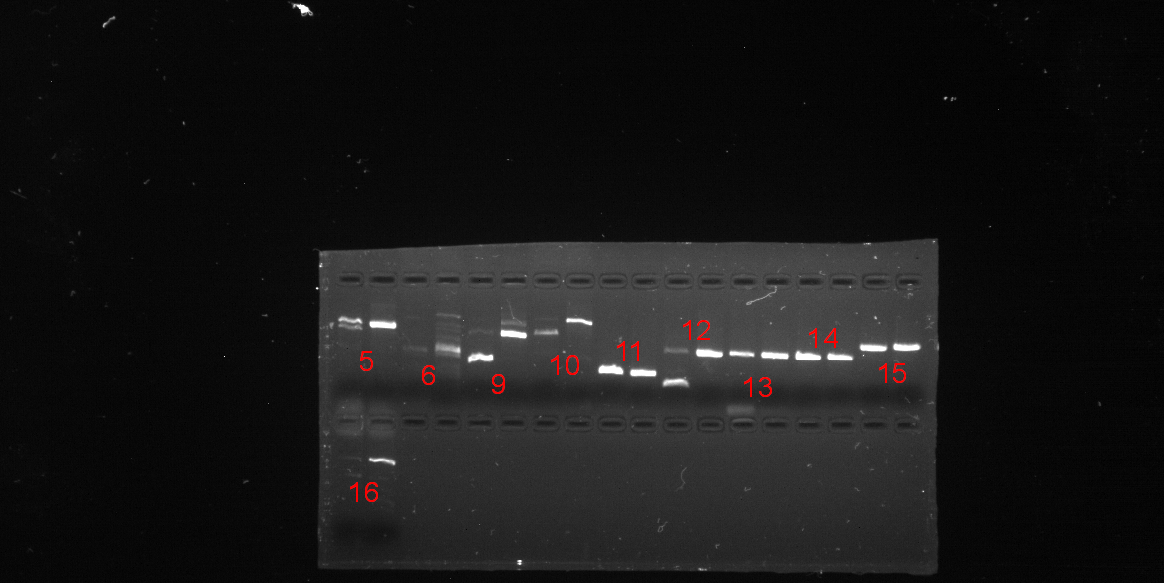

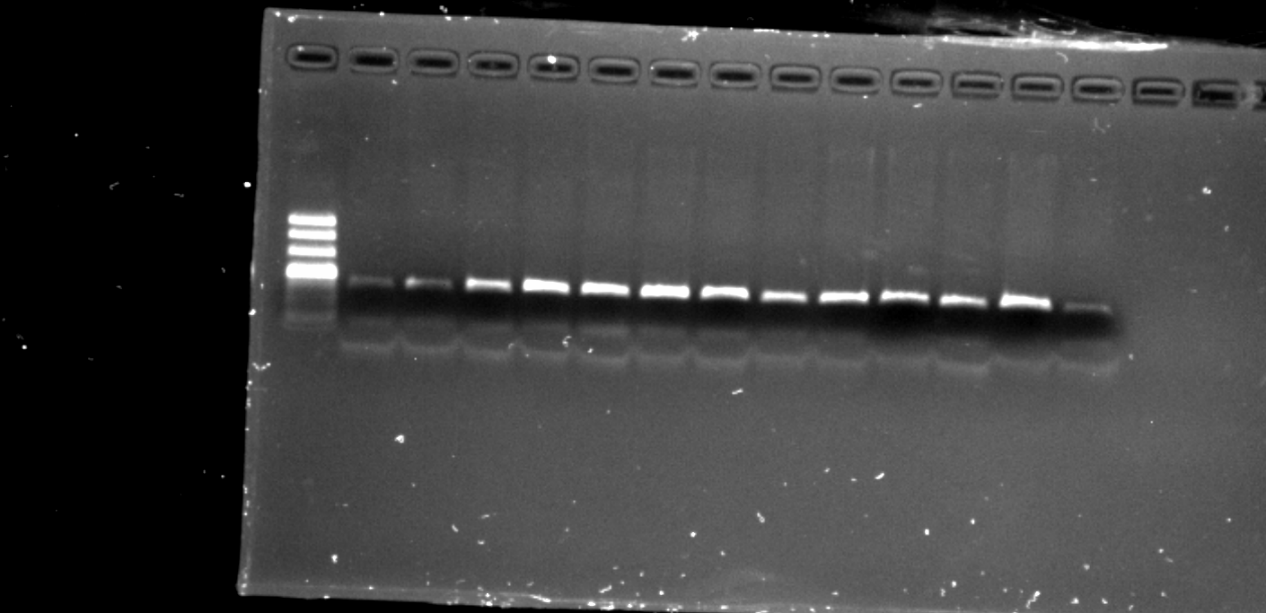


17


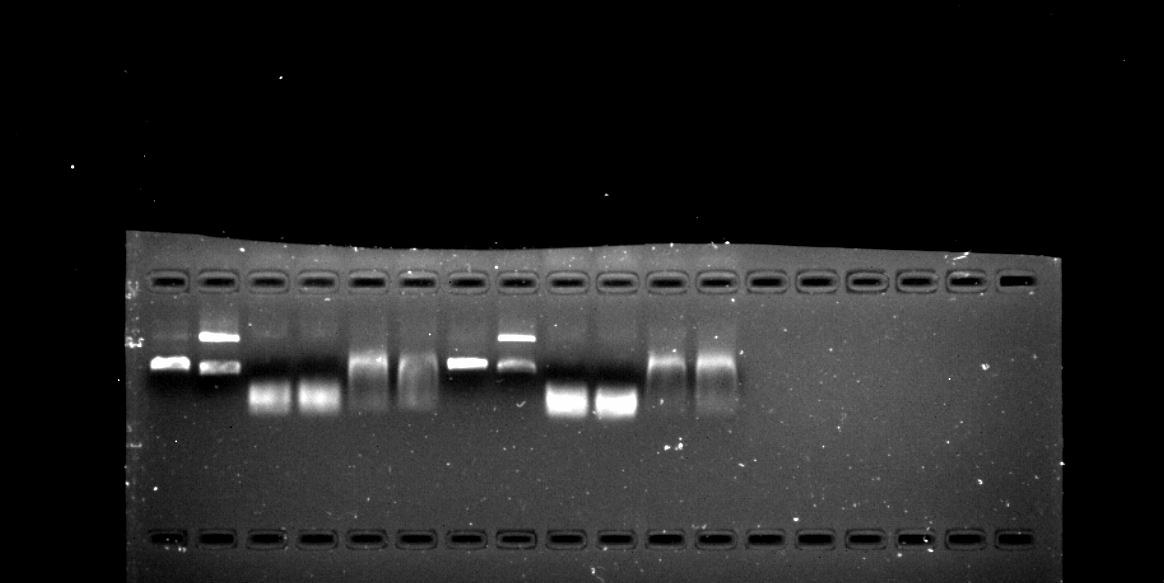


5 6


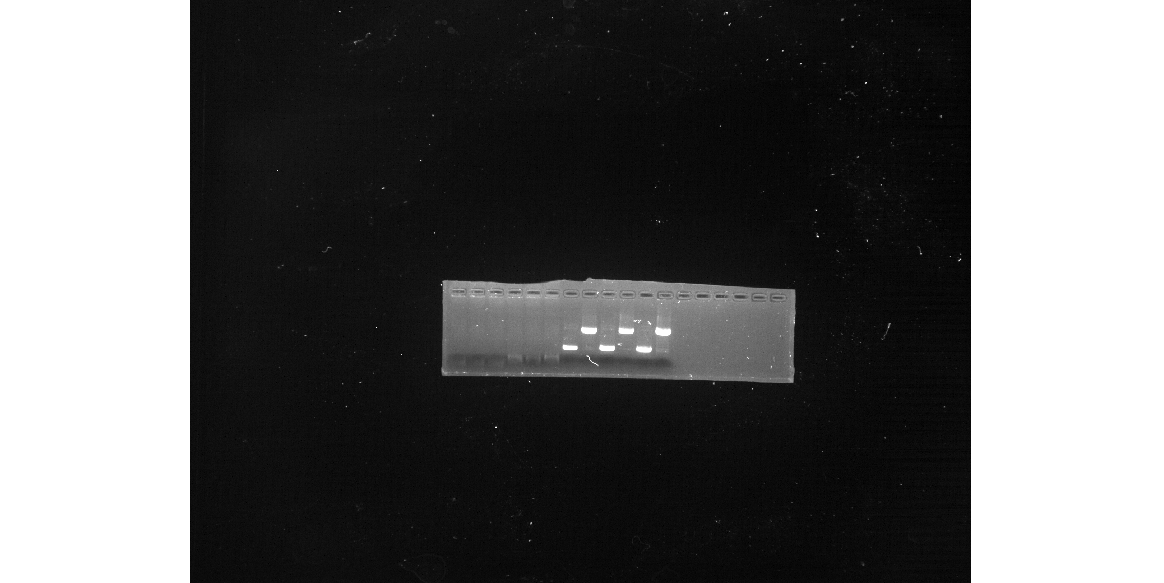


16

Figure S1. Original gel images for Figure 3. The 1-17 red boxes indicate genes of rps16, trnA, rpl2, petD, ndhB, trnK, trnL, trnV, ycf3, ndhA, rpl16, rps12, trnG, petB, trnI, atpF, and 23S, respectively. The red box contain WT and mutant, Left represents the WT, and the right represents the mutant.
