# Supplementary material for: OsCAF2 contains two CRM domains and is necessary for chloroplast development in rice
Source: BMC Plant Biol. 2020 Aug 18;20:381. doi: 10.1186/s12870-020-02593-z (PMC7437035; doi:10.1186/s12870-020-02593-z)
Supplement: Supplementary file 3 — Additional file 3: Figure S2. Original images for Fig. 4b. [file 12870_2020_2593_MOESM3_ESM.docx]

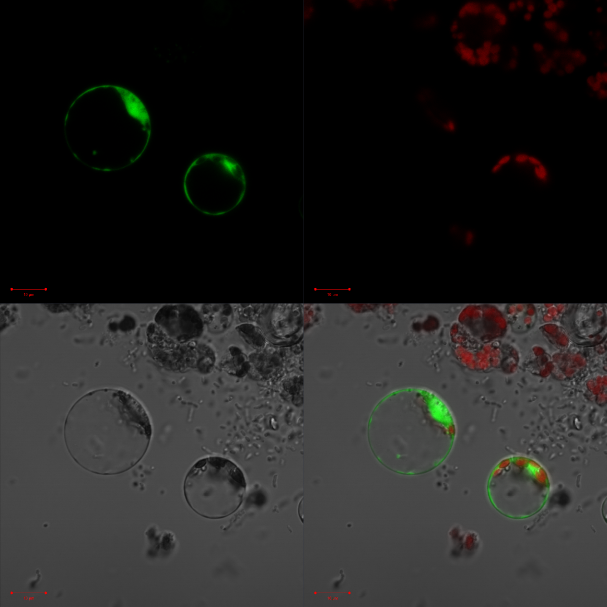

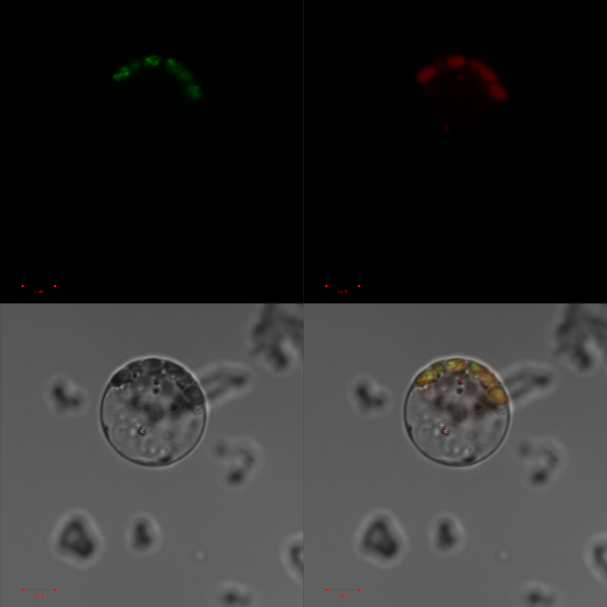


A

B

Figure S2. Original images for Figure 4b. a Subcellular localization of GFP protein. b Subcellular localization of GFP-OsCAF2 protein.
