# Supplementary material for: OsCAF2 contains two CRM domains and is necessary for chloroplast development in rice
Source: BMC Plant Biol. 2020 Aug 18;20:381. doi: 10.1186/s12870-020-02593-z (PMC7437035; doi:10.1186/s12870-020-02593-z)
Supplement: Supplementary file 5 — Additional file 5: Figure S4. Original images for Fig. 6. [file 12870_2020_2593_MOESM5_ESM.docx]

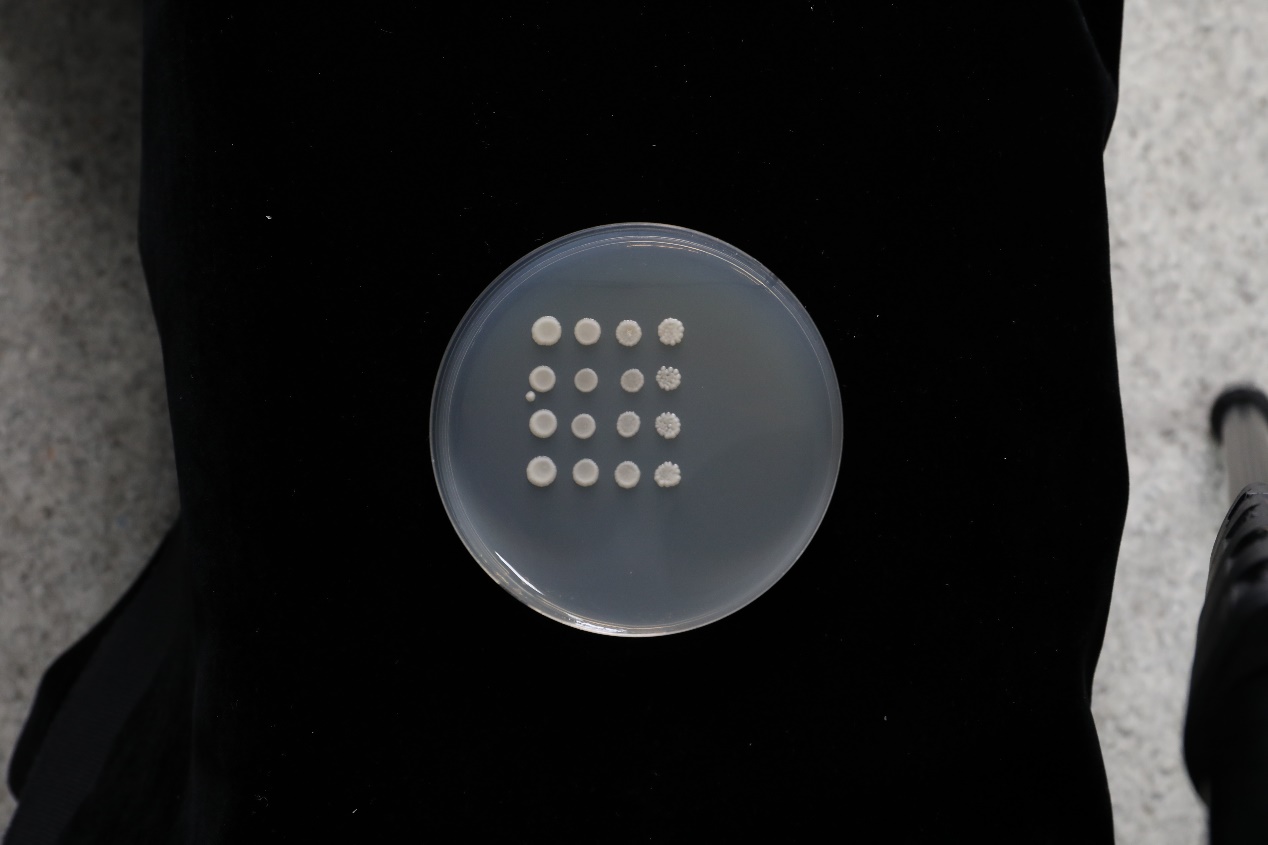

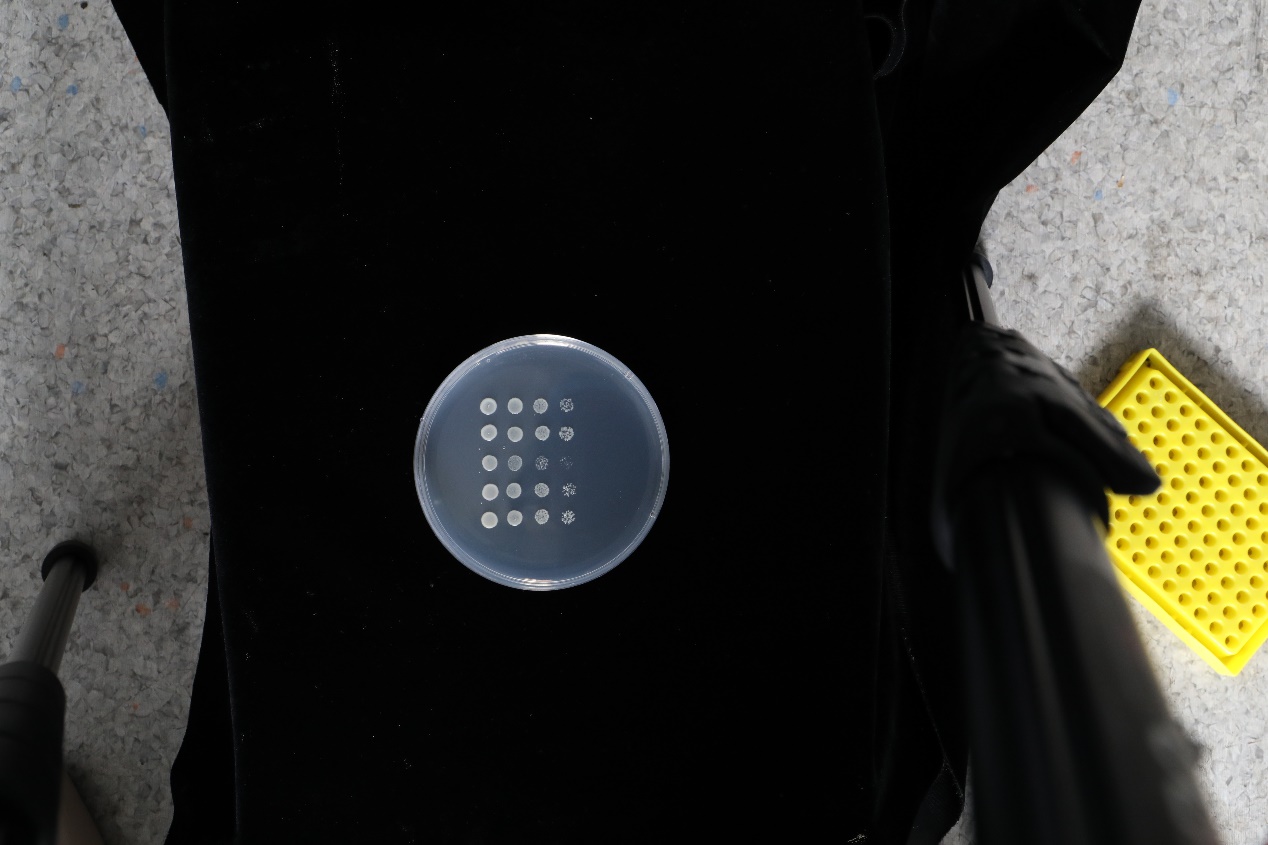

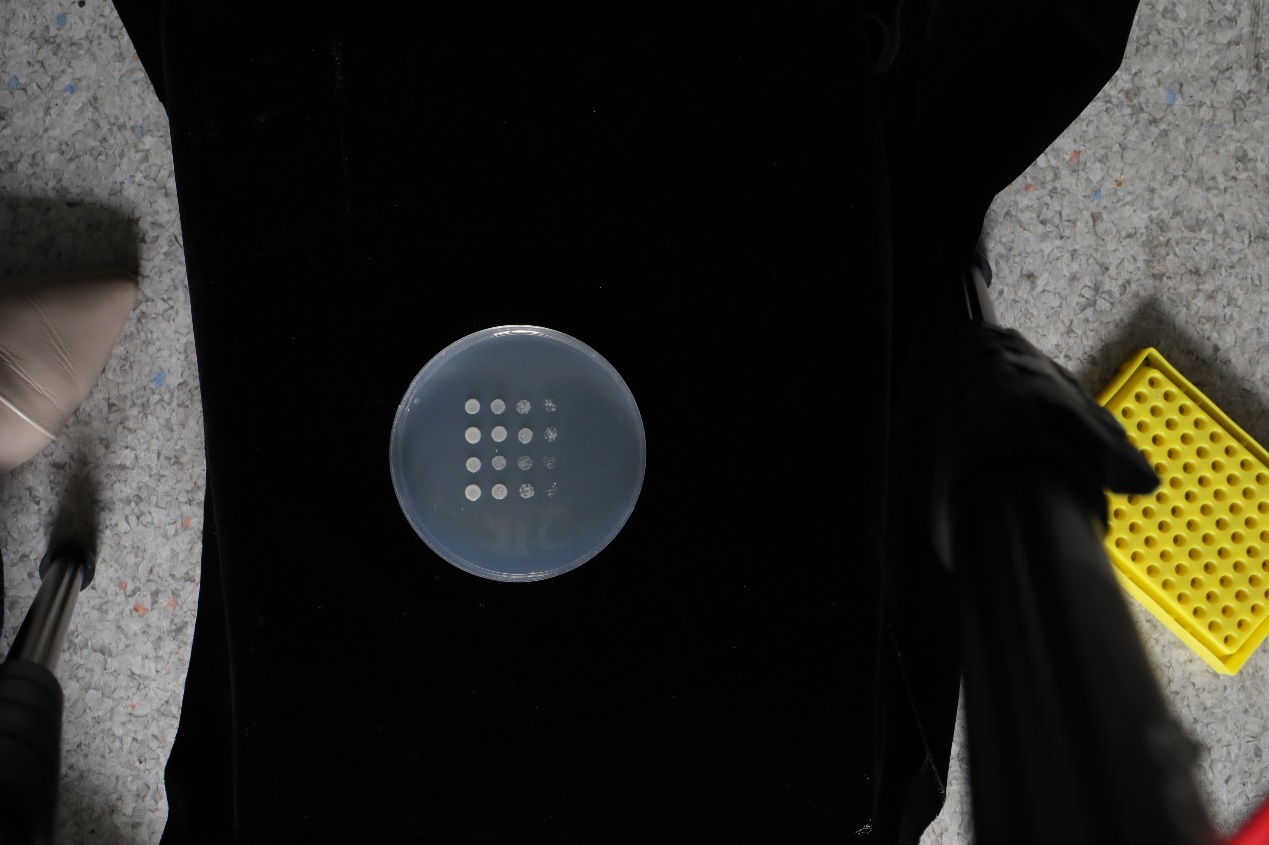

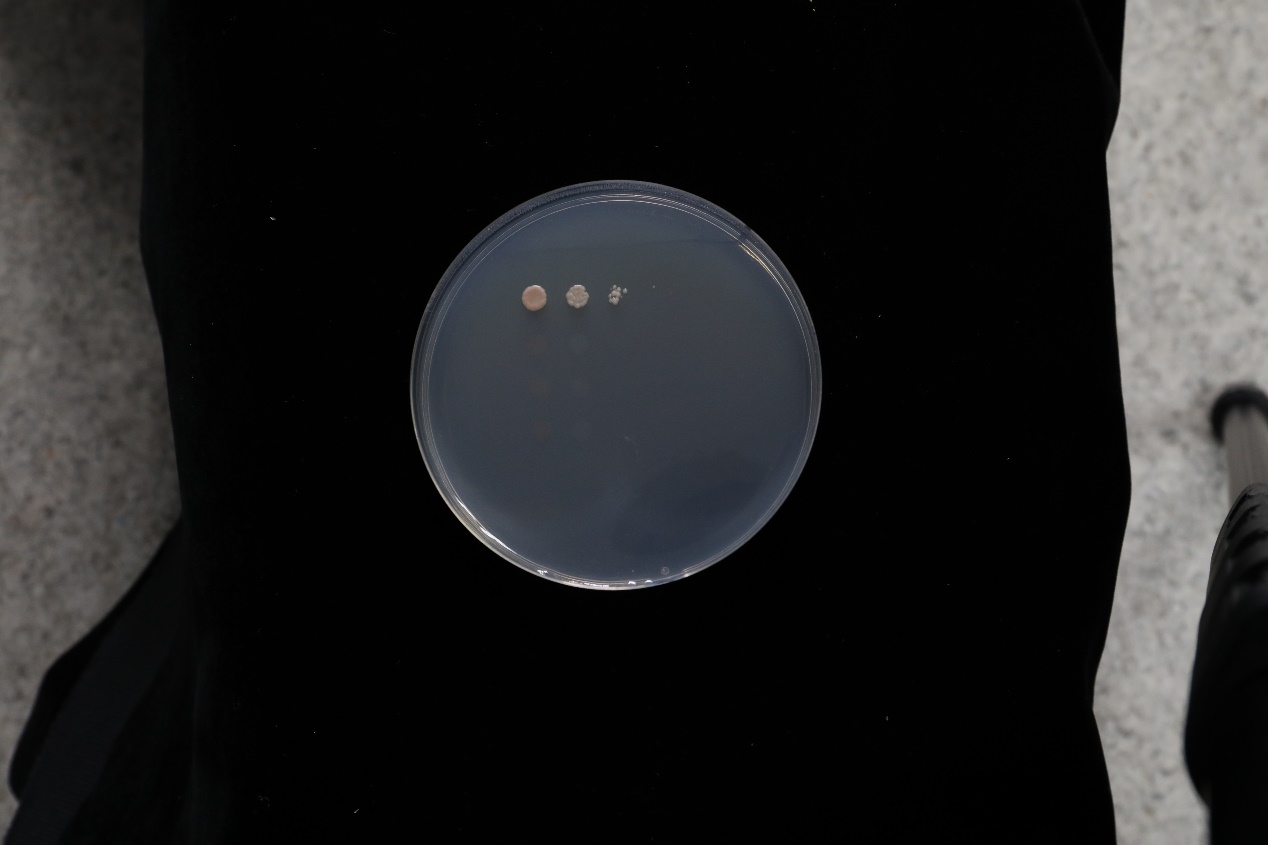

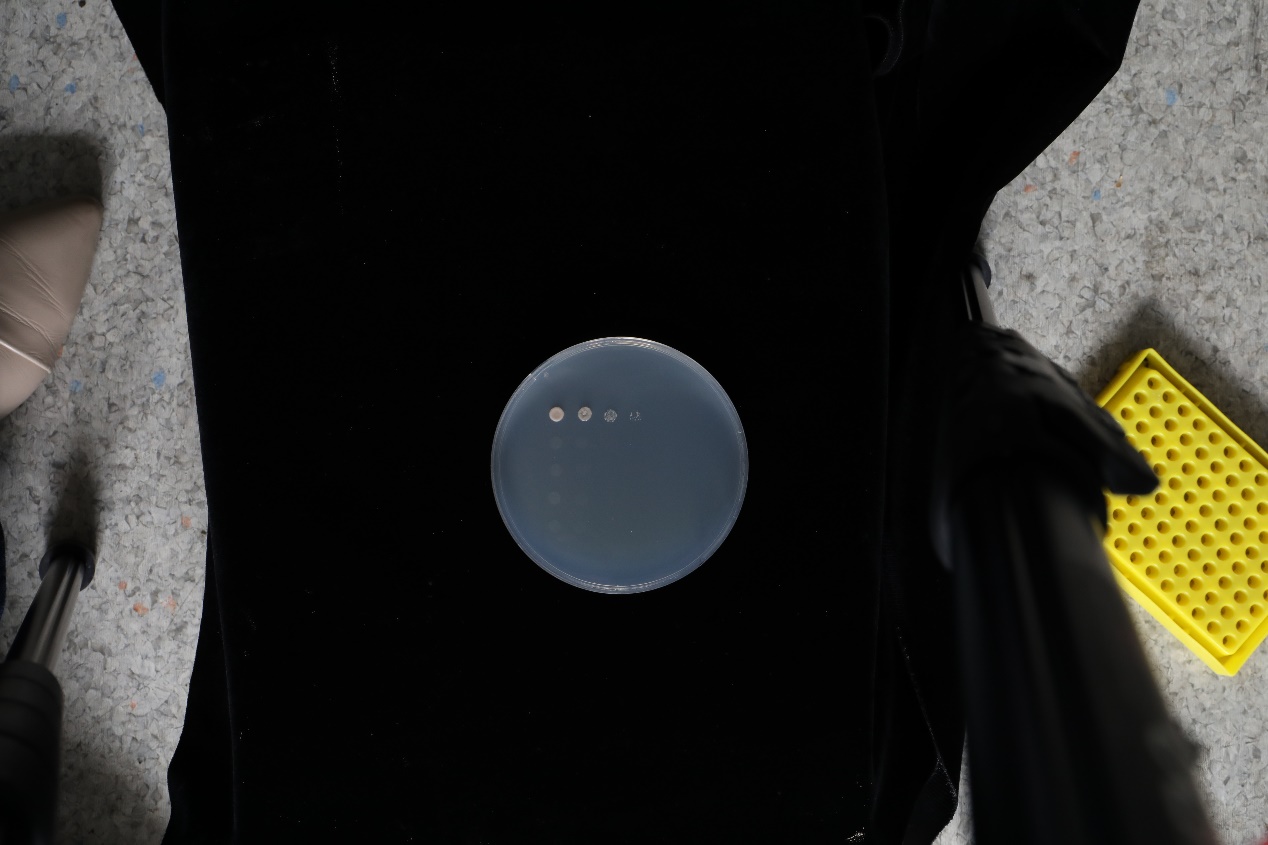

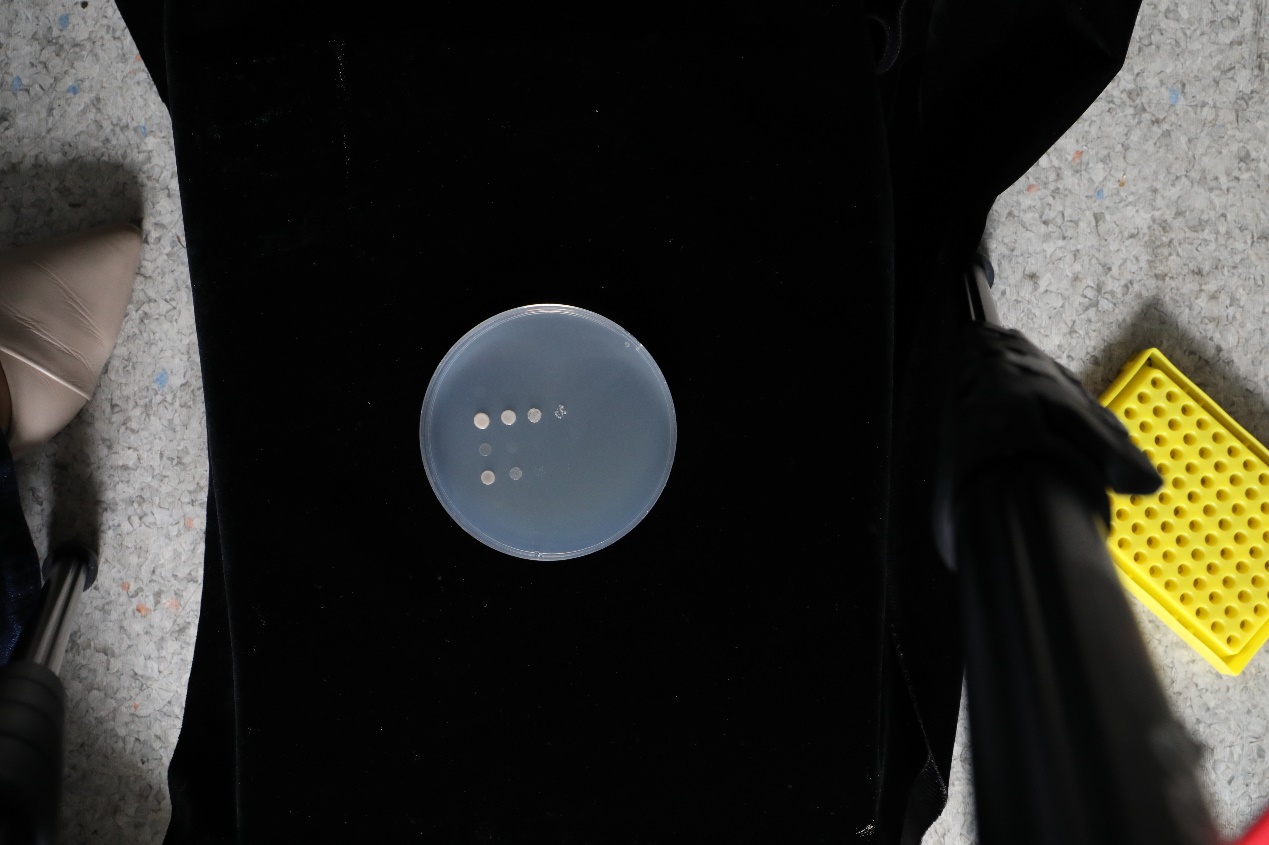


A

B

C

D

E

F

AD- OsCAF2+BD- OsCAF2

AD +BD- OsCAF2

AD- OsCAF2+BD

AD- OsCAF2-N+BD- OsCAF2-N

AD- OsCAF2-N+BD- OsCAF2-M

AD- OsCAF2-N+BD- OsCAF2-C

AD- OsCAF2-M+BD- OsCAF2-M

AD- OsCAF2-M+BD- OsCAF2-C

AD- OsCAF2-C+BD- OsCAF2-C

Figure S4. Original images for Figure 6. A, C, E were the SD-T/L media, B, D, F were the SD-/T/L/H/A media.
